# Hemostasis, coagulation and thrombin in venoarterial and venovenous extracorporeal membrane oxygenation: the HECTIC study

**DOI:** 10.1038/s41598-021-87026-z

**Published:** 2021-04-12

**Authors:** Bruce Cartwright, Hannah M. Bruce, Geoffrey Kershaw, Nancy Cai, Jad Othman, David Gattas, Jacqueline L. Robson, Sarah Hayes, Hayden Alicajic, Anna Hines, Alice Whyte, Nophanan Chaikittisilpa, Timothy James Southwood, Paul Forrest, Richard J. Totaro, Paul G. Bannon, Scott Dunkley, Vivien M. Chen, Mark Dennis

**Affiliations:** 1 Sydney Medical School, University of Sydney, Sydney, Australia; 2 Department of Cardiology, Royal Prince Alfred Hospital, Missenden Road, Camperdown, Sydney, NSW 2050 Australia; 3 Intensive Care Service, Royal Prince Alfred Hospital, Sydney, Australia; 4 Institute of Academic Surgery, Royal Prince Alfred Hospital, Sydney, Australia; 5 Department of Cardiothoracic Surgery, Royal Prince Alfred Hospital, Sydney, Australia; 6 Department of Anaesthetics, Royal Prince Alfred Hospital, Sydney, Australia; 7 Department of Haematology, Royal Prince Alfred Hospital, Sydney, Australia; 8 ANZAC Research Institute, University of Sydney, Sydney, Australia; 9 Department of Haematology, Concord Repatriation General Hospital, Sydney, Australia

**Keywords:** Heart failure, Platelets, Thrombosis

## Abstract

Extracorporeal membrane oxygenation (ECMO) support has a high incidence of both bleeding and thrombotic complications. Despite clear differences in patient characteristics and pathologies between veno-venous (VV) and veno-arterial (VA) ECMO support, anticoagulation practices are often the same across modalities. Moreover, there is very little data on their respective coagulation profiles and comparisons of thrombin generation in these patients. This study compares the coagulation profile and thrombin generation between patients supported with either VV and VA ECMO. A prospective cohort study of patients undergoing VA and VV ECMO at an Intensive care department of a university hospital and ECMO referral centre. In addition to routine coagulation testing and heparin monitoring per unit protocol, thromboelastography (TEG), multiplate aggregometry (MEA), calibrated automated thrombinography (CAT) and von-Willebrand’s activity (antigen and activity ratio) were sampled second-daily for 1 week, then weekly thereafter. VA patients had significantly lower platelets counts, fibrinogen, anti-thrombin and clot strength with higher d-dimer levels than VV patients, consistent with a more pronounced consumptive coagulopathy. Thrombin generation was higher in VA than VV patients, and the heparin dose required to suppress thrombin generation was lower in VA patients. There were no significant differences in total bleeding or thrombotic event rates between VV and VA patients when adjusted for days on extracorporeal support. VA patients received a lower median daily heparin dose 8500 IU [IQR 2500–24000] versus VV 28,800 IU [IQR 17,300–40,800.00]; < 0.001. Twenty-eight patients (72%) survived to hospital discharge; comprising 53% of VA patients and 77% of VV patients. Significant differences between the coagulation profiles of VA and VV patients exist, and anticoagulation strategies for patients of these modalities should be different. Further research into the development of tailored anticoagulation strategies that include the mode of ECMO support need to be completed.

## Introduction

Patients on ECMO exhibit a range of hemostatic changes including consumption of coagulation factors, thrombocytopenia, altered von Willebrand factor (vWF) multimers and platelet dysfunction^1^ and reductions in anti-thrombin levels^2^. Significant bleeding events occur in more than 30% of patients on ECMO^3^ and better control of anticoagulation may improve patient outcomes^4^. Thrombotic complications occur in up to 8–17% of patients on ECMO^5^. Both bleeding and thrombotic complications are associated with increased mortality^4,6,7^.

Whilst many clinicians assume significant differences in haemostatic and coagulation profiles of veno-arterial (VA ECMO) and veno-venous (VV ECMO) extracorporeal support exist, there is extremely limited data on their respective coagulation profiles and, as yet, thrombin generation in these modalities has not been compared. Moreover, despite the assumed differences, the anticoagulation protocols and monitoring applied to both ECMO modalities is often the same across modalities. We sought to describe the haemostatic changes and circuit parameters in VV and VA ECMO patients using conventional coagulation tests and thrombinography (CAT) for thrombin generation, thromboelastography (TEG)—including Platelet Mapping and Multiple Electrode Aggregometry (Multiplate) in order to better inform subsequent anticoagulation management in these patients.

## Materials and methods

### Study population and ethics approval

A single centre, prospective cohort study at Royal Prince Alfred Hospital, a university hospital and ECMO referral centre for New South Wales, Australia. Local ethics approval was obtained for this study No: X16-0407.

All ECMO patients were included in the study, unless they had one of the following exclusion criteria: prior commencement of therapeutic anticoagulation, pre-existing indication for therapeutic anti-coagulation, (e.g. atrial fibrillation, pulmonary embolism) pre-existing thrombotic or bleeding disorder, were aged < 16 years or were pregnant.

### Haemostatic management

Unfractionated heparin was titrated to an activated partial thromboplastin time (aPTT) between 60 to 80 s (changed to 50 to 70 s in February 2018). The decision to change the target aPTT range was made by intensive care unit in response to data pertaining to safety of lower dose heparin regimes. There was no change in aPTT assay used during the study and the upper normal range of the assay is 37 s. aPTT was monitored 4-hourly until target values had been reached then 6th hourly. Red blood cell (RBC) transfusion was given to maintain a hemoglobin (Hb) concentration above 80 g/L. Blood factor products, antifibrinolytic agents (tranexamic acid and antithrombin concentrate, fresh frozen plasma was administered when deemed clinically appropriate. ECMO circuits (HLS, Cardiohelp and PLS, Rotaflow, Getinge, Germany) were recombinant human albumin and heparin coated (Maquet—Bioline). Circuits were changed if there was evidence of systemic fibrinolysis presumed to be due to circuit or oxygenator on the basis of deteriorating oxygenator function, increases in trans-oxygenator pressure, rising d-dimer, plasma free haemoglobin and/or lactate dehydrogenase (LDH). Management of bleeding and thrombotic complications were at the discretion of the treating clinician.

### Blood sampling and laboratory analysis

All patients received at least daily standard pathology tests that included full blood count, serum biochemistry including: Electrolytes, Urea, Creatinine, Calcium, Magnesium, Phosphate, Albumin, Liver function tests (protein/GGT/ALP/AST/ALT), international normalised ratio (INR), aPTT and heparin Anti-FXa, fibrinogen, d-dimer, LDH and plasma free haemoglobin. Arterial blood gas analysis was completed at least twice daily. Second daily assessment of anti-thrombin, vWF Antigen and Activity, platelet aggregometry (ADPtest and TRAPtest—Multiplate), thromboelastography (TEG, including Platelet Mapping). Calibrated automated thrombinography (CAT) using the Stago Genesia DrugScreen application containing > 5 pM tissue factor as trigger was completed for the first week of ECMO support and weekly thereafter with concurrent APTT and anti-FXa recorded. All tests were completed one day post decannulation. Ventilatory parameters, hemodynamics, circuit variables, circuit clots and vasopressor requirements were monitored and logged hourly or more often if required.

### Bleeding and thrombosis definitions and data collection

Bleeding events were defined according to the Bleeding Academic Research Consortium (BARC) criteria^8^. Routine lower limb venous dopplers were completed post decannulation from ECMO.

### Statistical analysis

Baseline comparisons were performed using chi-square or Fisher’s exact test for dichotomous variables, Student t tests for normally distributed continuous variables and Mann-U Whitney tests otherwise with results reported as n (%), mean (SD), or median (inter-quartile range [IQR]), respectively. Pre-defined subgroup analyses included VV and VA ECMO. For pathology tests performed more than once per day, the highest, lowest and mean results for each day were recorded and used in the analysis. Comparisons between cohorts for daily blood tests were performed using linear or logistic mixed models with each subject modelled as a random effect, to account for repeated measures within patients. Log transformation was applied to skewed data where appropriate.

The incidence of bleeding and thrombotic events was calculated using cumulative incidence functions with death or decannulation as the competing risk, with cohorts compared using Gray’s test.

Analysis was performed using R statistical software version 3.5.2 (R Core Development Team, Vienna, Austria^9^) and a two-sided p value of 0.05 was considered to be statistically significant.

### Ethics approval and consent to participate

The study protocol conforms to the ethical guidelines of the 1975 Declaration of Helsinki and was approved by the Human Research and Ethics Review Committee of Sydney Local Health District (reference X16-0407) with approval for waiver on consent.

## Results

**Figure 1:**
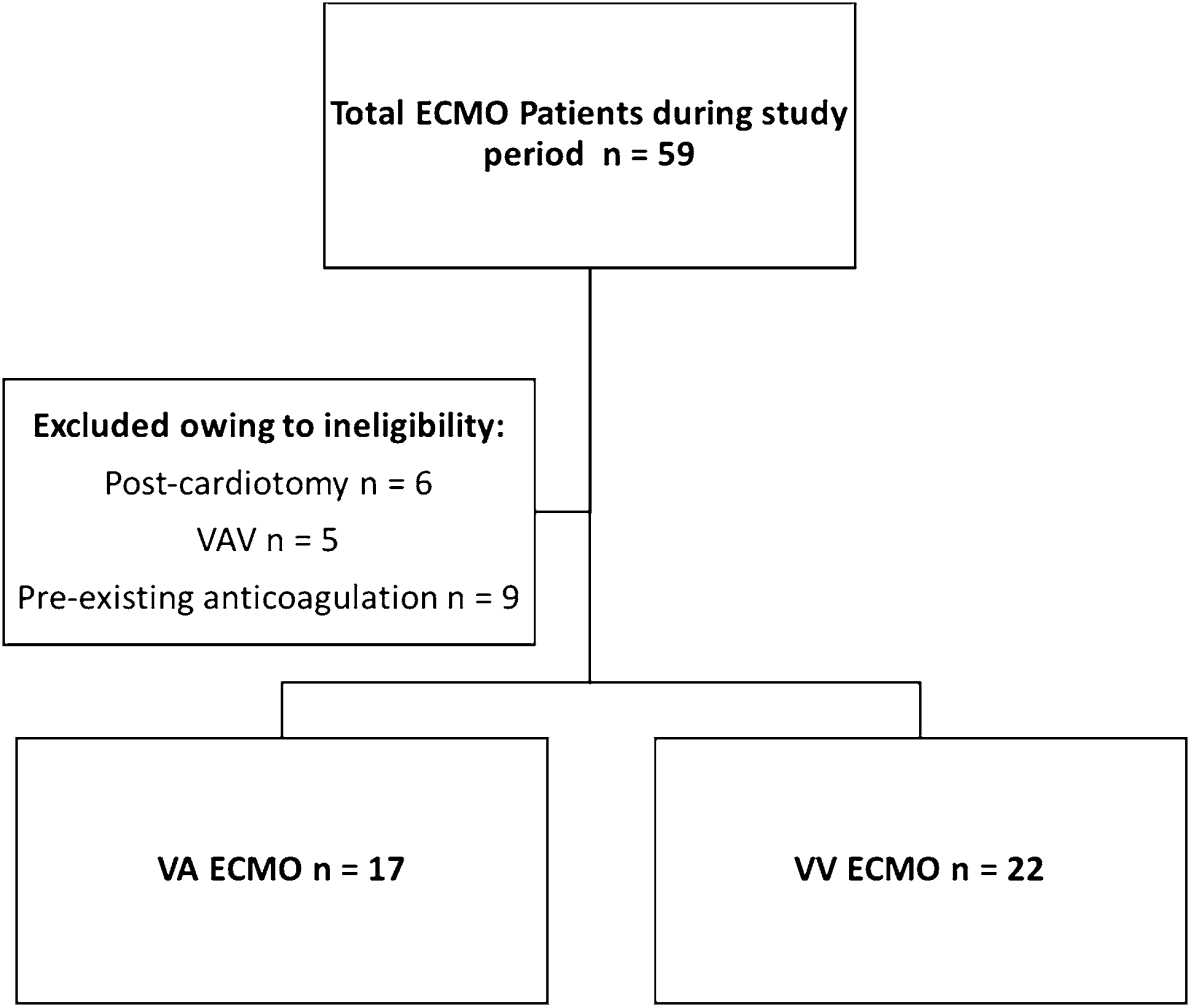
Total ECMO patients within study period and included patients.

Fifty-six patients required ECMO support during the study period (July 2017 to December 2019), (26 VV, 5 VAV and 25 VA). After exclusion—Fig. 1, 22 VV and 17 VA, median age 47 years (IQR 41–64); 23 (59%) male, were included. There were no COVID-19 cases. Baseline cohort characteristics are given in Table 1 and Table S1. No patient had pre-existing bleeding or thrombosis. Ten (59%) of the VA patients were extracorporeal cardiopulmonary resuscitation (ECPR) patients. Median Sequential Organ Failure Assessment score was VA 9 (IQR 6–12) and VV 8 (IQR 5–10); *p* = 0.255. VA patients had significantly higher pre-ECMO lactate 8.6 mmol/L (IQR 5.7–11.6) versus 1.1 mmol/L (IQR 0.8–1.9); *p* < 0.001, lower base excess and worse ventricular function; *p* < 0.001—Table S1. The overall median duration of support was 3 days (IQR 2–5) for VA versus VV 10 days (IQR 6–13); *p* < 0.001. Twenty-eight (72%) survived to ICU discharge; VA 9 (53%) versus VV 17 (77%); *p* = 0.482.

**Table 1:** Patient characteristics and heparin targets.

Variable (patients)	Overall (n = 39)	VA ECMO (n = 17)	VV ECMO (n = 22)	P value
Age, years, median (IQR)	46.7 (41.1, 63.7)	48.2 (40.8, 64.4)	46.35 (41.80, 61.33)	0.977
Male gender, n (%)	23 (59.0)	10 (58.8)	13 (59.1)	1.000
Body mass index, (kg/m^2^), median (IQR)	27.6 (23.5, 32.6)	25.9 (23.2, 30.1)	29.1 (23.7, 34.4)	0.257
Chronic respiratory condition, n (%)	5 (12.8)	0 (0.0)	5 (22.7)	0.056
Chronic liver disease, n (%)	2 (5.1)	0 (0.0)	2 (9.1)	0.495
History of ischaemic heart disease, n (%)	7 (17.9)	5 (29.4)	2 (9.1)	0.205
History of diabetes mellitus, n (%)	2 (5.1)	0 (0.0)	2 (9.1)	0.495
**Respiratory requiring VV ECMO**^**$**^**, n (%)**				
Bacterial pneumonia			3 (14.3)	
Viral pneumonia			8 (38.1)	
Aspiration pneumonia/pneumonitis			2 (9.5)	
Asthma			2 (9.5)	
Pulmonary insufficiency secondary to trauma			1 (4.8)	
Other			5 (22.7)	
**Cardiovascular diagnoses requiring VA ECMO, n (%)**				
Acute myocardial infarction		2 (16.7)		
Refractory arrhythmia		4 (33.3)		
Viral myocarditis		3 (25.0)		
Other		8 (47.0)		
**Pre-ECMO Cardio-respiratory status, n (%)**				
Cardiopulmonary resuscitation prior to ECMO		10 (58.8)		
Pre-ECMO Intra-aortic balloon pump	1 (2.6)	1 5.9)	0 (0.0)	0.436
Continuous renal replacement therapy	6 (15.4)	1 (5.9)	5 (22.7)	0.206
**Days of antiplatelet agent usage, n (%)**	**Overall (n = 221)**	**VA ECMO (n = 76)**	**VV ECMO (n = 145)**	
Any antiplatelet	34 (15.7)	17 (23.0)	17 (12.0)	0.690
Single antiplatelet	13 (5.9)	3 (3.9)	10 (6.9)	0.559
Dual antiplatelet	21 (9.5)	14 (18.4)	7 (4.7)	0.002
Aspirin	26 (11.8)	17 (22.4)	9 (6.2)	0.590
Clopidogrel	28 (12.7)	13 (17.1)	15 (10.3)	0.690
Ticagrelor	1 (0.5)	1 (1.3)	0 (0.0)	> 0.99
Tirofiban	1 (0.5)	1 (1.3)	0 (0.0)	> 0.99
**Heparin target ranges**				
No infusion	20 (9.4)	15 (20.5)	5 (3.8)	0.490
Activated partial thromboplastin time, (s) < 45	6 (2.9)	3 (4.1)	3 (2.3)	
Activated partial thromboplastin time, (s) 50–70	109 (52.9)	27 (36.5)	74 (55.6)	
Activated partial thromboplastin time, (s) 60–80	71 (34.5)	20 (27.4)	51 (38.3)	
Other	29 (13.7)	17 (23.0)	12 (8.7)	
**Heparin delivery (daily)**				
Daily heparin dose, IU* (median [IQR])	24,000.0 [9500.0, 36,000.0]	8500.0 [2500.0, 24,000.0]	28,800.0 [17300.0, 40,800.0]	< 0.001
Daily heparin dose, IU/kg/hr (median [IQR])	12.46 [6.82, 16.67]	9.59 [2.05, 14.04]	13.64 [8.74, 19.01]	0.004
Heparin infusion, hours per day (median [IQR])	24.00 [14.00, 24.00]	14.00 [4.50, 24.00]	24.00 [24.00, 24.00]	< 0.001

### Anticoagulation and biochemistry

Daily blood tests are summarised in Table 2 (full details—Tables S2 and S3). VA patients received significantly lower daily heparin doses compared to VV patients (8500 IU vs 28,800 IU; *p* < 0.001), heparin dose IU/kg/hr (median [IQR]) VA 9.59 [IQR 2.05, 14.04], VV 13.64 [IQR 8.74, 19.01]; *p* = 0.004. The median daily hours on heparin was 14 h [IQR 4.50, 24.00] versus VV 24 h [IQR 24.00, 24.00]; *p* < 0.001. There was no significant difference in the overall mean daily aPTT, however VA patients had significantly lower mean daily anti-FXa levels (0.06 IU/mL vs 0.27 IU/mL; *p* < 0.017. The daily mean aPTT was within the specified range for 90 (49%); VA 20 days (35%) versus VV 70 days (55%); *p* < 0.032—Table S3. One or more aPTT levels of greater than 100 s were recorded on 36 days with 29 (81%) occurring in the first 24 h of ECMO support. Anticoagulation was ceased more often in VA patients; 48 days versus VV 36 days; *p* < 0.001. VA patients had higher mean bilirubin, INR, d-dimer and lower fibrinogen and antithrombin—Table S2. Fibrinogen levels over the first week of ECMO support are depicted in Figure S1.

**Table 2:** Daily first week conventional coagulation and biochemical tests.

Laboratory test, median [IQR*]	Overall (n = 221)	VA ECMO (n = 76)	VV ECMO (n = 145)	P value
Mean bilirubin, (μmol)	11.0 [7.0, 27.5]	26.0 [13.0, 54.0]	9.00 [6.00, 17.1]	0.026
Mean lactate, (mmol/L)	1.4 [1.1, 2.00]	2.2 [1.2, 5.3]	1.25 [1.00, 1.6]	< 0.001
Mean lactate dehydrogenase, (units/L)	496.0 [294.0, 855.8]	879.5 [478.3, 2301.8]	427.50 [292.5, 627.5]	0.021
Mean hemoglobin, (g/L)	87.0 [79.5, 98.0]	88.0 [80.5, 102.5]	86.50 [79.5, 97.00]	0.548
Mean platelet count, (10^9^/L)	162.5 [87.3, 222.0]	97.0 [71.0, 146.0]	192.00 [134.0, 241.0]	0.161
Mean activated partial thromboplastin time, (s)	59.0 [49.0, 70.8]	59.5 [47.0, 79.0]	58.50 [49.5, 68.0]	0.119
Mean international normalised ratio	1.3 [1.1, 1.6]	1.8 [1.3, 2.3]	1.20 [1.10, 1.30]	< 0.001
Mean d-dimer, (mg/L)	2.2 [1.1, 6.8]	7.0 [1.8, 10.0]	1.59 [0.96, 4.04]	0.027
Mean fibrinogen, (g/L)	4.8 [3.1, 6.4]	2.6 [1.9, 4.4]	5.60 [4.28, 7.00]	< 0.001
Mean anti-Xa, (IU^!^/mL)	0.2 [0.1, 0.4]	0.1 [0.00, 0.2]	0.27 [0.11, 0.43]	0.017
Antithrombin III, (%)	68.0 [48.3, 85.3]	42.0 [33.5, 55.0]	78.5 [59.0, 90.3]	< 0.001
^%^vWF antigen, (%)	414.0 [344.0, 485.0]	420.5 [336.0, 490.0]	405.0 [353.0, 474.0]	0.271
vWF activity, (%)	239.0 [188.0, 342.5]	219.00 [195.5, 308.5]	259.0 [187.5, 347.5]	0.760
vWF ratio	0.6 [0.5, 0.7]	0.60 [0.56, 0.70]	0.6 [0.5, 0.8]	0.972

### Monitoring heparin dosing

**Figure 2:**
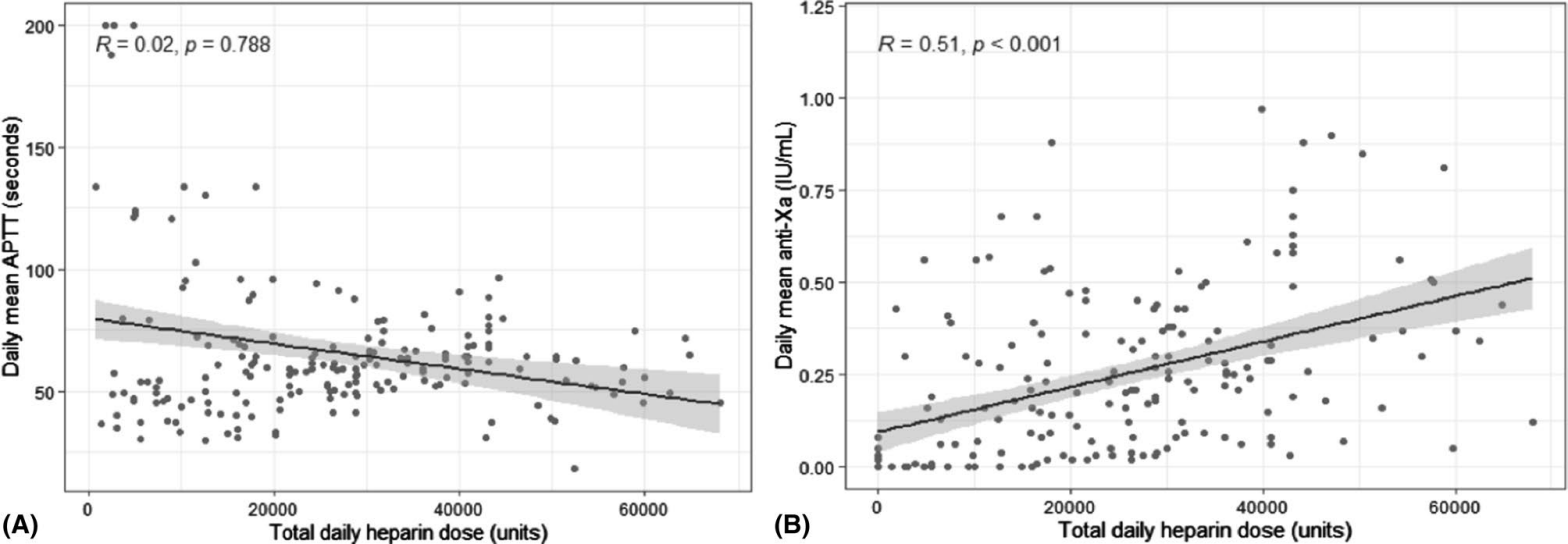
Total daily heparin dose on all ECMO patients demonstrating no significant relationship to (**A**) APTT and a significant relationship to (**B**) Anti-FXa levels.

There was a stronger correlation between median heparin dose over 24 h and anti-FXa compared to aPTT. There was no significant correlation between the mean aPTT with overall heparin dose, r = 0.02, *p* = 0.78 (samples without heparin excluded), while correlation with anti-FXa demonstrated r = 0.51, *p* < 0.001—Fig. 2A,B. In samples with matched anti-FXa and aPTT, VV patients demonstrated a lower aPTT for equivalent anti-FXa level compared with VA patients—Figure S2. APTT levels on Day zero were higher in VA patients (88.1 s (IQR 53.5–200.0) compared to VV 51.5 (IQR 40.1–63.1); *p* = 0.011, but anti-FXa levels were not different 0.26 (IQR 0.00–0.46) versus 0.13 (0.03–0.25); *p* = 0.707—Table S4.

### Thrombin generation: calibrated automated thrombinography

**Figure 3:**
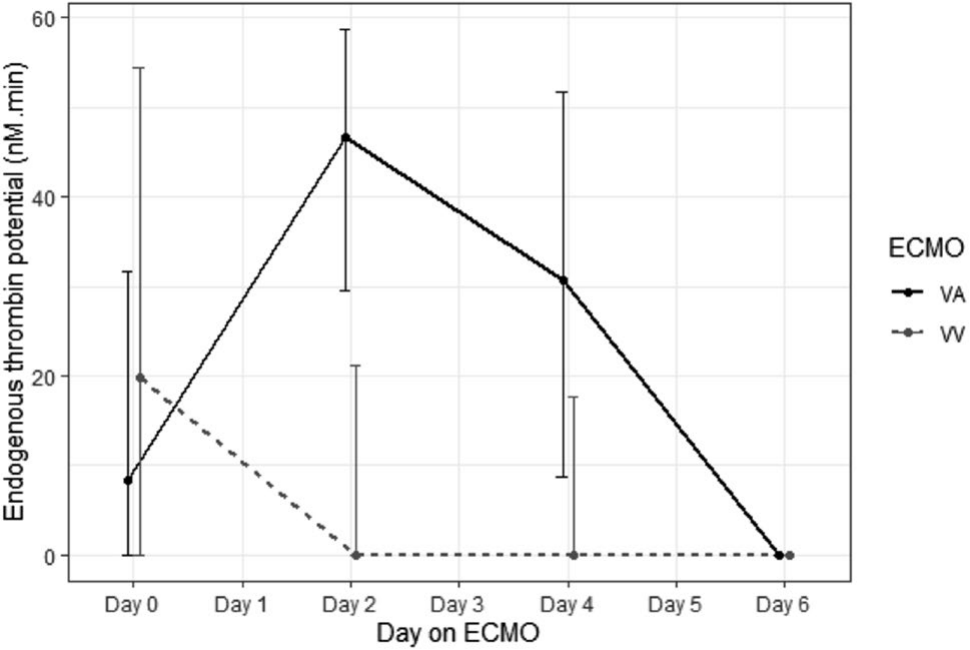
Endogenous Thrombin Potential (ETP) levels over first week of ECMO support.

**Figure 4:**
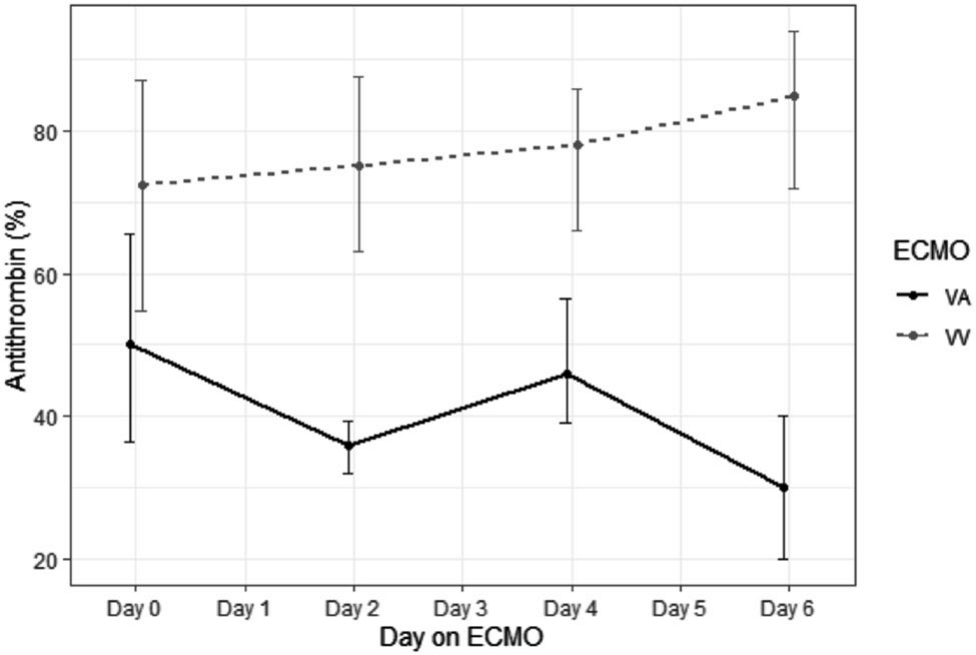
Antithrombin percentage over first week of ECMO support.

VA patients demonstrated overall shorter lag time, a longer time to peak and elevated endogenous thrombin potential (ETP)*—*Table S4. On samples where patients anti-FXa was subtherapeutic (< 0.30 IU/mL), 14 (39%) of VV patients did not have measurable thrombin generation (ETP of 0) compared versus 2 (10%) VA patient samples. i.e. > 90% of VA patients had residual thrombin generation with subtherapeutic anti-FXa versus 61% of VV patients (*p* = 0.038). In patient samples with complete thrombin suppression (ETP of 0), there was a significant difference in the median aPTT measured at the time of thrombin suppression with a wide range of aPTT values for VA patients; VA 200 s (IQR 98.8–200) vs VV 62.6 s (IQR 56.8, 70.4); *p* < 0.001. Endogenous thrombin potential (ETP) reduced throughout the first week of ECMO support, at day 6 there was no ETP in either VA or VV patients– Fig. 3. Corresponding antithrombin percentages over the first week is shown in Fig. 4.

Anti-FXa levels were more strongly correlated with markers of residual thrombin generation (negative correlation with peak thrombin and endogenous thrombin potential and positive correlation with both lag time and time to peak)—Table 3. aPTT and anti-FXa correlated with peak thrombin, ETP and time to peak in VA patients. Anti-FXa but not aPTT correlated with these thrombin generation parameters in VV patients.

**Table 3:** APTT and Anti-FXa correlation to markers of thrombin generation.

APTT	All		VA		VV	
	R	p	R	p	R	p
Peak Height, (nmol/L)	− 0.54	0.001	− 0.67	0.002	− 0.38	0.068
Endogenous thrombin potential, (nmol/L min)	− 0.44	0.003	− 0.67	0.002	− 0.27	0.203
Time to peak, (s)	0.26	0.086	0.45	0.048	0.13	0.539
Lag time, (s)	0.07	0.671	0.12	0.609	2	0.910

Anti-FXa	All		VA		VV	
	R	p	R	p	R	p
Peak Height, (nmol/L)	− 0.42	0.005	− 0.53	0.017	− 0.62	0.001
Endogenous thrombin potential, (nmol/L min)	− 0.55	< 0.001	− 0.64	0.002	− 0.63	0.001
Time to peak, (s)	0.55	< 0.001	0.61	0.004	0.45	0.031
Lag time, (s)	0.41	0.006	0.41	0.069	0.38	0.076

s = seconds.

### Thromboelastography (TEG)

Full TEG variables are summarised in Table S5. VA ECMO patients had significantly lower R-time on CK-R 15.4 min (IQR 8.5–30.9) versus VV 26.2 min (IQR 15.6–42.0); *p* = 0.013 but not on CKH VA 9.4 (IQR 8.4–2.10) versus VV 10.8 (IQR 8.3–13.1); *p* = 0.235. VA ECMO patients had lower median CKH A10 and functional fibrinogen; *p* < 0.001.

### Platelet function and clot strength

Median platelet count was lower in the VA group 97 mm^3^/L (IQR 71–146) versus 192 mm^3^/L (IQR 134–241); *p* < 0.001 and platelet counts declined throughout ECMO run—Figure S3. There was no significant difference in median vWF antigen, activity or vWF activity to antigen ratio—Table S1. VA patients had significantly lower indices of clot strength on TEG Platelet Mapping compared to VV patients when measured by TEG and multiplate multi-platelet aggregometry—Table S6). There was no significant difference in days on any antiplatelets; VA 17 (22%) versus VV 17 (12%); 0.690, nor number of patients who had any antiplatelet: VV 3 patients (13.6%) versus VA 4 patients (23.5%); *p* = 0.677. VA patients had significantly more days on dual antiplatelet therapy VA 14 (18%) days versus VV 7 (5%); *p* = 0.002. On patient days without any anti-platelet therapy VA patients had a lower ADP result 21 aggregometry units (IQR 14–72.5) versus VV 64 (IQR 25.5–100.0); *p* = 0.035 with non-significant trend on TRAP, 71 (IQR 54.0–131.5) versus VV 125 (IQR 100.5–162.3); *p* = 0.052.

### Bleeding and thrombotic events

Twenty-five bleeding events occurred in total (16 VV, 9 VA) amongst 15 patients (32%)—Table S7. Two fatal bleeding events occurred, both in VV patients. Bleeding occurred earlier in VA patients (0.30 versus VV—4.30 days, *p* = 0.001). There was no significant difference in the cumulative incidence of bleeding events nor thrombotic events—Figure S4 and Figure S5 respectively*.* VA ECMO patients also had more days with blood product usage and greater total use of packed red blood cells, fresh frozen plasma, and cryoprecipitate. A total of 26 thrombotic events occurred (7 VA ECMO, 19 VV ECMO); *p* < 0.002. Twelve of the 17 (63%) thrombotic events on VV ECMO patients were circuit changes or clots, seven were deep vein thrombosis noted on post decannulation venous ultrasonography.

### Blood product and factor supplementation

Blood product utilisation is provided in Table S2. A median number of 2 packed red blood cells (PRBC) [IQR [0.00, 4.00] was given per patient. There was no difference in amount of blood product supplementation between VA and VV cohorts. The total number of platelets transfused for VA patients was 10, VV patients 16, and on days when platelets were transfused there was no difference in quantity transfused VA 2 units (IQR 1.25–2.00) versus VV 1 (IQR 1–2.50); *p* = 0.777. No antithrombin concentrate was supplemented during the study.

### Circuit variables

ECMO circuit and cannulation details are outlined in Table S8 and S9. VV patients had higher transmembrane pressure gradients compared to VA patients.

### VA ECMO subgroup analysis

Additional subgroup analyses were completed comparing non-ECPR VA patients with ECPR patients and then VV patients—Table S10 and S11. When compared to non-ECPR VA patients, ECPR patients had significantly higher lactate 6.2 (IQR 2.5–8.9) versus 1.6 (IQR 1.20–2.92); *p* = 0.028, lower fibrinogen 2.2 (IQR 1.63–2.40) versus 4.15 (IQR 2.62–5.88); *p* = 0.014 and lower vWF activity 204 (IQR 288–226) versus 341 (IQR 219–375); *p* = 0.017.

Non-ECPR VA patients had higher lactate, bilirubin, lactate dehydrogenase, INR and d-dimer with no difference in fibrinogen when compared to VV patients. Antithrombin level was significantly lower when compared to VV patients—Table S11.

## Discussion

ECMO patients exhibit a range of coagulation abnormalities including: consumption of both procoagulant and anticoagulant factors^10,11^, thrombocytopenia, altered vWF multimers^12^, platelet dysfunction^1^, decreased anti-thrombin^2^, as well as increased D-dimer, prothrombin fragment 1.2 and thrombin-antithrombin complexes^13^. Despite patients who require VA and VV ECMO support having substantially different characteristics and underlying pathologies, data on differences in underlying coagulation profiles is extremely limited and patients are often treated with the same anticoagulation protocols. Herein, using multiple measures of coagulation, we report the largest and most detailed study to date on the coagulation profiles in VA and VV ECMO patients, and confirm substantial differences between VA and VV coagulation profiles suggesting that coagulation management should be considered different between the two modalities.

A pattern of consumptive coagulopathy was present in VA patients; characterised by elevated d-dimers, bilirubin and lactate and prolonged prothrombin time and lower fibrinogen and platelet levels, decreased clot strength and platelet dysfunction. Further, VA patients received lower doses of heparin than VV patients to achieve similar aPTT levels, and had longer CK-R time but shorter CKH-R times by thromboelastography. These findings indicate that VA ECMO patients have higher consumption of clotting factors than VV patients, and that aPTT is a less reliable monitor of heparin therapy than in this group.

The differences in coagulation profile between VA and VV patients is likely in large part, to be explained by the differing baseline patient characteristics and underlying pathologies requiring support. VA patients with inherent cardiac dysfunction and periods of poor end-organ perfusion and liver dysfunction prior to, and during implementation of extracorporeal support may exhibit decreased coagulation factor production^14^. Moreover, 10 of our 19 VA patients received ECPR for cardiac arrest. Cardiac arrest results in systemic inflammation, increased coagulation factor consumption, disseminated intravascular coagulation (DIC), induction of tissue factor-dependent coagulation, impaired anticoagulant mechanisms^15,16^ and increased fibrinolysis^16^. Whilst the large proportion of ECPR patients in the VA group may contributed to some of differences when compared to VV patients, comparing non-ECPR VA patients to VV patients on subgroup analysis, differences in key parameters remained, thus supporting underlying differences between VA and VV patients independent of cardiac arrest status. Whilst cardiogenic shock with organ failure and a consumptive process appears to predominate in VA patients, ARDS patients typically exhibit an extreme inflammatory response with diffuse fibrin deposition^17^, a pro-coagulant response^18^ and massive thrombin generation^17^—differing underlying pathological processes.

Interestingly, significant differences were also seen between ECPR and non-ECPR patients in fibrinogen levels and lactate levels. This may be due to a more pronounced acute coagulative response in arrest patients.

We found lower indices of clot strength in with VA patients compared to VV patients with lower TEG Maximum Amplitude (MA); and lower G-value (a marker of overall platelet and fibrin performance). Low clot strength is predictive of bleeding events^19^ and a TEG CK G value below < 5 dynes/cm^2^ (as found in in our VA patients) is associated with increased risk of hemorrhage^20^. Our testing could not determine the relative contribution of hypofibrinogenemia, anti-platelet medication, intrinsic platelet dysfunction, and abnormal vWF on the abnormal clot strength.

Whilst hypofibrinogenemia and transient reduction in fibrinogen levels^21^ have been reported, many studies report normal or supranormal fibrinogen levels during ECMO^19,21,22^ (as we report in our VV patients) with an acute phase reaction from systemic inflammation in ARDS patients a likely contributor^16^. The decreased clot strength we found in our VA patients may have been caused by hypofibrinogenemia due to increased consumption and was most pronounced in ECPR patients presumably due to increased consumptive or fibrinolytic processes^16^.

We assessed platelet function with TEG Platelet Mapping and Multiplate Aggregometry and found that VA ECMO patients had significantly lower ADP activity and a trend to lower activity on TRAPtest results. ECMO related platelet dysfunction assessed by Multiplate aggregometry has been described previously in VV and VA ECMO patients^23–25^. Proposed mechanisms for this include: depletion of the stored platelet ADP^26^, sheer flow induced shedding of platelet adhesion glycoproteins^27^, loss of high molecular weight VWF multimers (HMWM) with reduced vWF activity^28–30^ and lower levels of platelet aggregation^27,28,31,32^. However, thrombocytopenia influences platelet function testing and some studies^24,25^ have not corrected for this. Balle et al. found no dysfunction compared to controls when results were corrected for platelet levels^33^. Thrombocytopenia, seen in our study, is common in both ECMO modalities^34–36^. Pre-ECMO platelet levels and development of critical illness may contribute to the its development^37^ more than duration of ECMO support and this may explain our finding of lower platelet count in VA compared to VV patients.

Relative thrombin generation in ECMO patients is only just being elucidated. Only one previous study of thrombin generation in VA patients has been reported^38^. Our study, is the first to look at residual thrombin generation in the presence of heparin effect during standard care in ECMO patients and demonstrates differences between VA and VV cohorts in response to UFH. Despite the expected large thrombin generation seen in ARDS patients on VV, our data suggested that VV patients were more likely to achieve thrombin suppression with lower anti-FXa levels than VA. The overall increased thrombin generation found in our VA patients likely reflect lower total heparin doses and lower anti-thrombin levels when compared to VV patients. Anti-thrombin deficiency, especially within the initial days of support, is commonly reported in ECMO patients^39^ and is suspected to be caused by due to activation of coagulation, impaired synthesis, increased fibrinolysis and disseminated intravascular coagulation^40^. Future studies may be able to elucidate if this is related to variation in anti-thrombin consumption.

In heparinised patients demonstrating residual thrombin generation activity, anti-FXa levels, but not APTT, correlated with classical markers of thrombin generation indicating that anti-FXa may offer a more accurate method of guiding heparin dose, highlighted by the high variability in APTT at similar thrombin generation levels seen in the VA patients. In combination with our data showing the poor correlation between total heparin dose and aPTT, these results add to the literature suggesting that a randomised comparison between anti-FXa and aPTT as methods for adjusting UFH dosing in ECMO may be warranted. Clinical adjustments to monitoring UFH and changes to target ranges may also need to take in account inherent differences in VV versus VA patients in relationships between anti-FXa, aPTT levels and residual thrombin generation.

In our study only about one half of aPTT values were within prescribed range. This number is lower than documented in previous prospective randomised studies^41^ but higher than other cohort studies^42^ and reflects the difficulty in accurately titrating heparin in ECMO patients. VA patients had a higher proportion of aPTTs above the therapeutic range despite lower total heparin dosage and anti-FXa levels. A majority of very high aPTT (> 100 secs) values occurred in the first 24 h after initiation of ECMO support (especially in VA cases), most likely when these patient’s coagulation profiles are most deranged, and when they are more likely to have received bolus heparin doses for interventions (such as coronary angiography). High mean aPTT and level at 24 h post ECMO cannulation has been shown to be predictive of bleeding events^4,43^. Our finding that all major VA bleeding events within this time, (compared to Day 4 on VV ECMO patients) reinforces the need for meticulous anticoagulation management during this time.

## Limitations

The aPTT target range was changed during the study, to a slightly lower APTT target.

We did not manage bleeding or thrombotic complications (including circuit changes) by pre-defined protocol. Ultrasonic screening for thrombotic complications was only performed following decannulation and therefore thrombotic complications may have been identified later that when they occurred. Intensive monitoring of variables was limited to the first week of ECMO support and weekly thereafter, it is possible on after the first week coagulation parameters may continue to change. Our VA group included a significant number of ECPR cases. Whilst we did perform subgroup analysis comparing non-ECPR, ECPR and VV ECMO patients, a larger number of patients is needed to adequately characterise and compare these cohorts. VV ECMO patients had substantially longer ECMO runs when compared VA and our intensive coagulation monitoring ceased at 1 week. It is possible the coagulation profile of VV patients was not adequately captured by this.

## Conclusions

Substantial differences in the coagulation and haemostatic profile of VA and VV patients exist and the monitoring using traditional anticoagulation tests, in particular aPTT, is highly variable and the heparin required to suppress thrombin generation varied between ECMO modality. Tailored anticoagulation protocols for and monitoring of VA and VV ECMO patients may be of benefit and randomized control trials of differing protocols should be attempted.

## Supplementary Information

Supplementary Information

## Data Availability

The datasets generated and/or analysed during the current study are not publicly available, owing to the clinical nature of the dataset, but are available from the corresponding author on reasonable request but would require additional ethical approval by Sydney Local Health District Research and Ethics Committee.
