# Supplementary material for: Hemostasis, coagulation and thrombin in venoarterial and venovenous extracorporeal membrane oxygenation: the HECTIC study
Source: Sci Rep. 2021 Apr 12;11:7975. doi: 10.1038/s41598-021-87026-z (PMC8042030; doi:10.1038/s41598-021-87026-z)
Supplement: Supplementary file 1 — Supplementary Information [file 41598_2021_87026_MOESM1_ESM.docx]

**Hemostasis, Coagulation and Thrombin in Venoarterial and Venovenous Extracorporeal Membrane Oxygenation - The HECTIC Study – Supplementary Data: Tables and Figures**

Bruce Cartwright^a,f^ MBBS, FANZCA; Hannah M Bruce BMed MD, BSci(Med)Hons^c^, Geoffrey Kershaw^g,h^ BSc, FAIMS; Nancy Cai BBioTech^g^; Jad Othman MBBS, FRACP, FRCPA^i^ ; David Gattas^a,c^ MBBS, MMed, FCICM, FRACP; Jacqueline L Robson MBBS (Hons)^f^ MMed FANZCA; Sarah Hayes MBBS, Hayden Alicajic MBBS, BMedSci (Hons 1)^c^ Anna Hines^f^ MBBS; Alice Whyte^f^ MBBS, FANZCA; Nophanan Chaikittisilpa^f^ MBBS; Timothy James Southwood MBBS MSc FCICM^c^, Paul Forrest^a,f^, MBChB, FANZCA; Richard J Totaro^a,c^ MBBS, FCICM, FRACP; Paul G Bannon^a,d,e^ MBBS, PhD, FRACS; Scott Dunkley MBBS, FRACP, [FRCPA](javascript:;)^a^; Vivien M. Chen^a,h,i^, MBBS, (Hons), PhD, FRACP, FRCPA; *Mark Dennis^a,b^, MBBS (Hons), PhD, FRACP

^a^Sydney Medical School, University of Sydney, Sydney, Australia

^b^Department of Cardiology, Royal Prince Alfred Hospital, Sydney, Australia

^c^Intensive Care Service, Royal Prince Alfred Hospital, Sydney, Australia

^d^Institute of Academic Surgery, Royal Prince Alfred Hospital, Sydney, Australia

^e^Department of Cardiothoracic Surgery, Royal Prince Alfred Hospital, Sydney, Australia

^f^Department of Anaesthetics, Royal Prince Alfred Hospital, Sydney, Australia

^g^Department of Haematology, Royal Prince Alfred Hospital, Sydney, Australia

^h^ANZAC Research Institute, University of Sydney, Sydney, Australia

^i^Department of Haematology, Concord Repatriation General Hospital, Sydney, Australia

*Full list of collaborating authors provided in supplementary online material

All authors take responsibility for all aspects of the reliability and freedom from bias of the data presented and their discussed interpretation.

**Corresponding Author and Reprints**

Dr Mark Dennis – ORCID: 0000-0002-1281-1324

Cardiology Department, Royal Prince Alfred Hospital

Missenden Road, Camperdown, NSW 2050 Australia

Ph: 61-2-9515 6111

Fax: 61-2-9519 4938

Email: mark.dennis@sydney.edu.au

**Financial Support:**

Nil.

**Conflict of Interest:**

None declared.

**Table S1 – Full Demographics and Pre-ECMO Status**

|  | Overall (n = 39) | | VA ECMO (n = 17) | | VV ECMO (n=22) | | p | |  |
| --- | --- | --- | --- | --- | --- | --- | --- | --- | --- |
| Age, median [IQR]* | | 46.70 [41.1, 63.7] | | 48.20 [40.8, 64.4] | | 46.35 [41.8, 61.3] | | 0.977 | |
| Male gender, n (%) | | 23 (59.0) | | 10 (58.8) | | 13 (59.1) | | 1.000 | |
| Body Mass Index, (kg/m^2^), median [IQR] | | 27.55 [23.6, 32.6] | | 25.86 [23.2, 30.1] | | 29.07 [23.7, 34.4] | | 0.257 | |
| Chronic respiratory condition, n (%) | | 5 (12.8) | | 0 (0.0) | | 5 (22.7) | | 0.056 | |
| Chronic liver disease, n (%) | | 2 (5.1) | | 0 (0.0) | | 2 (9.1) | | 0.495 | |
| Existing renal impairment, n (%) | | 1 (2.6) | | 0 (0.0) | | 1 (4.5) | | 1.000 | |
| Existing immunological condition, n (%) | | 2 (5.1) | | 0 (0.0) | | 2 (9.1) | | 0.495 | |
| Existing immunological treatment, n (%) | | 3 (7.9) | | 1 (5.9) | | 2 (9.5) | | 1.000 | |
| Background of Ischaemic heart disease, n (%) | | 7 (17.9) | | 5 (29.4) | | 2 (9.1) | | 0.205 | |
| Background of diabetes mellitus, n (%) | | 2 (5.1) | | 0 (0.0) | | 2 (9.1) | | 0.495 | |
| **Respiratory diagnoses requiring VV^&^ ECMO^$^, n (%)** | |  | |  | |  | |  | |
| Bacterial Pneumonia | |  | |  | | 3 (14.3) | |  | |
| Viral Pneumonia | |  | |  | | 8 (38.1) | |  | |
| Aspiration Pneumonia/Pneumonitis | |  | |  | | 2 (9.5) | |  | |
| Asthma | |  | |  | | 2 (9.5) | |  | |
| Pulmonary insufficiency secondary to trauma | |  | |  | | 1 (4.8) | |  | |
| Other | |  | |  | | 6 (27.3) | |  | |
| **Cardiovascular diagnoses required VA^#^ ECMO, n (%)** | |  | |  | |  | |  | |
| Acute myocardial infarction | |  | | 2 (16.7) | |  | |  | |
| Refractory arrhythmia | |  | | 4 (33.3) | |  | |  | |
| Viral myocarditis | |  | | 3 (25.0) | |  | |  | |
| Other | |  | | 8 (47.0) | |  | |  | |
| **Pre-ECMO Cardio-respiratory status, n (%)** | |  | |  | |  | |  | |
| Cardiopulmonary resuscitation prior to ECMO | | 10 (25.6) | | 10 (58.8) | | **-** | | <0.001 | |
| Pre-ECMO Intra-aortic balloon pump | | 1 (2.6) | | 1 5.9) | | 0 (0.0) | | 0.436 | |
| Continuous renal replacement therapy | | 6 (15.4) | | 1 (5.9) | | 5 (22.7) | | 0.206 | |
| Noradrenaline | | 22 (56.4) | | 11 (64.7) | | 11 (50.0) | | 0.517 | |
| Adrenaline | | 11 (28.2) | | 9 (52.9) | | 2 (9.1) | | 0.004 | |
| Milrinone | | 1 (2.6) | | 1 (5.9) | | 0 (0.0) | | 0.436 | |
| Vasopressin | | 3 (7.7) | | 2 (11.8) | | 1 (4.5) | | 0.570 | |
| Dobutamine | | 2 (5.1) | | 2 (11.8) | | 0 (0.0) | | 0.184 | |
| Metaraminol | | 1 (2.6) | | 0 (0.0) | | 1 (4.5) | | 1.000 | |
| Levosimenden | | 1 (2.6) | | 1 (5.9) | | 0 (0.0) | | 0.436 | |
| Pulmonary vasodilator | | 4 (10.5) | | 1 (5.9) | | 3 (14.3) | | 0.613 | |
| Prone positioning | | 3 (7.7) | | 0 (0.0) | | 3 (13.6) | | 0.243 | |
| Neuromuscular blockade | | 16 (41.0) | | 5 (29.4) | | 11 (50.0) | | 0.325 | |
| Corticosteroids | | 6 (15.4) | | 1 (5.9) | | 5 (22.7) | | 0.206 | |
| **Pre-ECMO Echocardiography, n (%)** | |  | |  | |  | |  | |
| *Left ventricular function* | |  | |  | |  | | <0.001 | |
| Normal | | 14 (35.9) | | 2 (11.8) | | 12 (54.5) | |  | |
| Hyperdynamic | | 8 (20.5) | | 0 (0.0) | | 8 (36.4) | |  | |
| Mild impairment | | 1 (2.6) | | 0 (0.0) | | 1 (4.5) | |  | |
| Moderate impairment | | 3 (7.7) | | 2 (11.8) | | 1 (4.5) | |  | |
| Severe impairment | | 12 (30.8) | | 12 (70.6) | | 0 (0.0) | |  | |
| *Right ventricular function* | |  | |  | |  | | <0.001 | |
| Normal | | 12 (30.8) | | 1 (5.9) | | 11 (50.0) | |  | |
| Hyperdynamic | | 6 (15.4) | | 0 (0.0) | | 6 (27.3) | |  | |
| Mild impairment | | 6 (5.4) | | 2 (11.8) | | 4 (18.2) | |  | |
| Moderate impairment | | 3 (7.7) | | 3 (17.6) | | 0 (0.0) | |  | |
| Severe impairment | | 11 (28.2) | | 10 (58.8) | | 1 (4.5) | |  | |
| *Pulmonary Hypertension* | |  | |  | |  | | 0.569 | |
| None | | 11 (28.2) | | 7 (41.2) | | 4 (18.2) | |  | |
| Mild | | 1 (2.6) | | 0 (0.0) | | 1 (4.5) | |  | |
| Moderate | | 4 (10.3) | | 1 (5.9) | | 3 (13.6) | |  | |
| Severe | | 3 (7.7) | | 1 (5.9) | | 2 (9.1) | |  | |
| **Pre-ECMO Ventilatory Status, median [IQR]** | |  | |  | |  | |  | |
| Fraction of inspired oxygen (FiO2), %, | | 100.00 [100.00, 100.00] | | 100.00 [78.00, 100.00] | | 100.00 [100.00, 100.00] | | 0.078 | |
| Positive end-expiratory pressure, (cmH_2_O) | | 12.00 [8.00, 15.00] | | 10.00 [6.50, 10.00] | | 13.50 [10.00, 17.25] | | 0.022 | |
| Peak inspiratory pressure, (cmH_2_O) | | 30.00 [25.00, 34.00] | | 25.00 [20.50, 26.00] | | 32.50 [30.00, 35.00] | | 0.001 | |
| Tidal volume, (mls) | | 400.00 [335.00, 450.00] | | 400.00 [367.50, 464.00] | | 400.00 [335.00, 450.00] | | 0.729 | |
| Ventilatory rate per minute | | 18.00 [14.00, 20.00] | | 14.00 [12.00, 16.00] | | 20.00 [15.75, 23.50] | | 0.008 | |
| Pao2:Fio2 ratio, (mmHg) | | 68.00 [58.50, 232.25] | | 290.00 [213.75, 363.00] | | 60.50 [57.00, 67.00] | | <0.001 | |
| Murray Score | | 3.00 [2.00, 3.38] | | 2.00 [1.25, 2.25] | | 3.25 [3.00, 3.75] | | <0.001 | |
| **Pre-ECMO Arterial Blood Gas, median [IQR]** | |  | |  | |  | |  | |
| pH. | | 7.19 [7.02, 7.33] | | 7.06 [6.96, 7.36] | | 7.24 [7.13, 7.29] | | 0.476 | |
| PaO_2,_ (mmHg) | | 68.00 [58.75, 142.75] | | 182.50 [105.25, 276.25] | | 60.00 [55.50, 67.00] | | <0.001 | |
| PaCO_2,_ (mmHg) | | 59.00 [36.00, 84.00] | | 34.00 [27.00, 53.50] | | 75.00 [56.75, 87.25] | | <0.001 | |
| HCO_3,_ (mmol/L) | | 22.00 [13.50, 25.00] | | 12.50 [11.00, 18.75] | | 25.00 [23.00, 27.00] | | <0.001 | |
| Base Excess | | -5.50 [-12.88, -1.82] | | -14.00 [-20.00, -9.00] | | -2.10 [-6.25, -0.30] | | <0.001 | |
| Lactate, (mmol/L) | | 2.85 [1.00, 7.00] | | 8.60 [5.65, 11.55] | | 1.10 [0.80, 1.90] | | <0.001 | |
| Arterial oxygen saturation, (%) | | 93.00 [88.00, 99.00] | | 99.00 [98.00, 99.00] | | 88.00 [83.25, 89.75] | | <0.001 | |
| **Antiplatelet Agent Usage, n (%)** | | **Overall (n = 221)** | | **VA ECMO (n = 76)** | | **VV ECMO (n = 145)** | | **P value** | |
| Days on any antiplatelet | | 34 (15.7) | | 17 (23.0) | | 17 (12.0) | | 0.690 | |
| Single antiplatelet | | 13 (5.9) | | 3 (3.9) | | 10 (6.9) | | 0.559 | |
| Dual antiplatelet | | 21 (9.5) | | 14 (18.4) | | 7 (4.7) | | 0.002 | |
| Aspirin | | 26 (11.8) | | 17 (22.4) | | 9 (6.2) | | 0.590 | |
| Clopidogrel | | 28 (12.7) | | 13 (17.1) | | 15 (10.3) | | 0.690 | |
| Ticagrelor | | 1 (0.5) | | 1 (1.3) | | 0 (0.0) | | >0.99 | |
| Tirofiban | | 1 (0.5) | | 1 (1.3) | | 0 (0.0) | | >0.99 | |
| **Heparin target range and dosage, n (%)** | | **Overall (n = 221)** | | **VA ECMO (n = 76)** | | **VV ECMO (n = 145)** | | **P value** | |
| No infusion | | 20 (9.4) | | 15 (20.5) | | 5 (3.8) | | 0.490 | |
| Activated partial thromboplastin time <45 seconds | | 6 (2.9) | | 3 (4.1) | | 3 (2.3) | |  |  |
| Activated partial thromboplastin time 50-70 seconds | | 109 (52.9) | | 27 (36.5) | | 74 (55.6) | |  |  |
| Activated partial thromboplastin time 60-80 seconds | | 71 (34.5) | | 20 (27.4) | | 51 (38.3) | |  |  |
| Other | | 29 (13.7) | | 17 (23.0) | | 12 (8.7) | |  | |
| **Heparin delivery (days)** | |  | |  | |  | |  | |
| Daily heparin dose, (IU), median [IQR] | | 24000.00 [9500.00, 36000.00] | | 8500.00 [2500.00, 24000.00] | | 28800.00 [17300.00, 40800.00] | | <0.013 | |
| Daily heparin dose, IU/kg/hr (median [IQR]) | | 12.46 [6.82, 16.67] | | 9.59 [2.05, 14.04] | | 13.64 [8.74, 19.01] | | 0.012 | |
| Heparin infusion, hours per day (median [IQR]) | | 24.00 [14.00, 24.00] | | 14.00 [4.50, 24.00] | | 24.00 [24.00, 24.00] | | <0.005 | |
| **First week conventional coagulation and biochemical tests, median [IQR]** | | | | | | | | | |
| Mean bilirubin, (μmol) | | 11.00 [7.00, 27.50] | | 26.00 [13.00, 54.00] | | 9.00 [6.00, 17.12] | | 0.026 | |
| Mean lactate, (mmol/L) | | 1.35 [1.05, 2.00] | | 2.15 [1.20, 5.30] | | 1.25 [1.00, 1.55] | | <0.001 | |
| Mean lactate dehydrogenase, (units/L) | | 496.00 [294.00, 855.75] | | 879.50 [478.25, 2301.75] | | 427.50 [292.50, 627.50] | | 0.021 | |
| Mean haemoglobin, (g/L) | | 87.00 [79.50, 98.00] | | 88.00 [80.50, 102.50] | | 86.50 [79.50, 97.00] | | 0.548 | |
| Mean platelet count, (10^9^/L) | | 162.50 [87.25, 222.00] | | 97.00 [71.00, 146.00] | | 192.00 [134.00, 241.00] | | 0.161 | |
| Mean aPTT, (s) | | 59.00 [49.00, 70.75] | | 59.50 [47.00, 79.00] | | 58.50 [49.50, 68.00] | | 0.119 | |
| Mean international normalised ratio | | 1.25 [1.10, 1.55] | | 1.75 [1.30, 2.30] | | 1.20 [1.10, 1.30] | | <0.001 | |
| Mean d-dimer, (mg/L) | | 2.17 [1.06, 6.81] | | 7.01 [1.79, 10.00] | | 1.59 [0.96, 4.04] | | 0.027 | |
| Mean fibrinogen, (g/L) | | 4.80 [3.10, 6.40] | | 2.60 [1.91, 4.38] | | 5.60 [4.28, 7.00] | | <0.001 | |
| Mean anti-Xa, (IU/mL) | | 0.21 [0.05, 0.37] | | 0.06 [0.00, 0.23] | | 0.27 [0.11, 0.43] | | 0.017 | |
| Antithrombin III, (%) | | 68.00 [48.25, 85.25] | | 42.00 [33.50, 55.00] | | 78.50 [59.00, 90.25] | | <0.001 | |
| ^%^vWF antigen, (%) | | 414.00 [344.00, 485.00] | | 420.50 [336.00, 490.00] | | 405.00 [353.00, 474.00] | | 0.271 | |
| vWF activity, (%) | | 239.00 [188.00, 342.50] | | 219.00 [195.50, 308.50] | | 259.00 [187.50, 347.50] | | 0.760 | |
| vWF ratio | | 0.62 [0.53, 0.73] | | 0.60 [0.56, 0.70] | | 0.64 [0.53, 0.76] | | 0.972 | |
| **Blood product and factor supplementation** | |  | |  | |  | |  | |
| *Blood product utilization in first week* | |  | |  | |  | |  | |
| Days on which any packed red blood cell given | | 56 (25.3) | | 26 (34.2) | | 30 (20.7) | | 0.190 | |
| Days on which any fresh frozen plasma given | | 14 (6.3) | | 13 (17.1) | | 1 (0.7) | | <0.001 | |
| Days on which any Cryoprecipitate given | | 9 (4.1) | | 9 (11.8) | | 0 (0.0) | | <0.001 | |
| Days on which any Platelets given | | 10 (4.5) | | 6 (7.9) | | 4 (2.8) | | 0.60 | |
| *Blood product utilization on per patient basis* | |  | |  | |  | |  | |
| Median number of packed red blood cells units per patient (total) | | 2.00 [0.00, 4.00] | | 3.00 [0.00, 4.00] | | 1.50 [0.00, 2.75] | | 0.455 | |
| Median number of fresh frozen plasma units per patient (total) | | 0.00 [0.00, 1.50] | | 2.00 [0.00, 3.00] | | 0.00 [0.00, 0.00] | | <0.001 | |
| Median number of cryoprecipitate units per patient (total) | | 0.00 [0.00, 0.00] | | 0.00 [0.00, 5.00] | | 0.00 [0.00, 0.00] | | <0.001 | |
| Median number of platelet units per patient (total) | | 0.00 [0.00, 0.00] | | 0.00 [0.00, 0.00] | | 0.00 [0.00, 0.00] | | 0.435 | |
| *Number of products transfused on days when blood products were transfused* | |  | |  | |  | |  | |
| PRBC per day on days where transfused | | 2.00 [1.00, 2.00] | | 2.00 [1.00, 2.00] | | 2.00 [1.00, 2.00] | | 0.696 | |
| FFP per day on days where transfused | | 2.00 [2.00, 4.00] | | 2.00 [2.00, 4.00] | | 1.00 [1.00, 1.00] | | 0.116 | |
| Cryoprecipitate per day on days where transfused | | 5.00 [5.00, 5.00] | | 5.00 [5.00, 5.00] | | NA | | NA | |
| Platelet per day, on days where transfused | | 2.00 [1.00, 2.00] | | 2.00 [1.25, 2.00] | | 1.00 [1.00, 2.50] | | 0.777 | |
| *Total number of Blood products transfused* | |  | |  | |  | |  | |
| Total number of packed red blood cells | | 100 | | 49 | | 51 | |  | |
| Total number of fresh frozen plasma units | | 43 | | 42 | | 1 | |  | |
| Total number of cryoprecipitate | | 56 | | 56 | | 0 | |  | |
| Total number of platelets | | 16 | | 10 | | 6 | |  | |

**IQR = Interquartile range, ^&^VV = Veno-venous, ^$^ECMO = Extracorporeal Membrane Oxygenation, ^#^VA = Veno-arterial, ^!^IU = International Unit, ^%^vWF = von-Willebrand’s Factor.*

*Data presented as medians (25–75% percentiles) unless otherwise specified*

**Table S2 – Daily standard lab tests first week**

| Standard lab tests for first week, median [IQR] | Overall (n = 221) | VA (n = 76) | VV (n = 145) | p |
| --- | --- | --- | --- | --- |
| Low ionised calcium, (mmol/L) | 1.11 [1.04, 1.17] | 1.07 [1.02, 1.13] | 1.12 [1.05, 1.20] | 0.022 |
| High lactate, (mmol/L) | 1.70 [1.20, 2.45] | 2.60 [1.40, 6.50] | 1.50 [1.20, 1.80] | <0.001 |
| High urea, (mmol/L) | 10.80 [7.50, 16.05] | 8.05 [5.23, 10.68] | 12.60 [9.20, 19.60] | 0.001 |
| High creatinine, (mmol/L) | 104.00 [82.00, 169.00] | 104.00 [85.25, 172.75] | 103.50 [77.00, 159.75] | 0.315 |
| High bilirubin (μmol) | 13.00 [7.00, 30.75] | 30.00 [16.00, 65.50] | 10.00 [7.00, 17.25] | 0.016 |
| High lactate dehydrogenase, (units/L) | 601.00 [329.00, 1408.00] | 1185.00 [601.00, 2936.25] | 440.00 [263.00, 672.00] | 0.021 |
| Low haemoglobin, (g/L) | 84.00 [77.00, 95.00] | 84.00 [76.00, 100.00] | 83.00 [77.00, 95.00] | 0.944 |
| Low platelet, (10^9^/L) | 149.50 [80.50, 208.75] | 89.00 [59.00, 130.00] | 186.00 [129.00, 225.00] | 0.093 |
| Plasma haemoglobin, (%) | 3.00 [2.50, 5.50] | 4.00 [2.50, 5.50] | 3.00 [2.50, 5.50] | 0.977 |
| High aPTT, (s) | 63.00 [53.00, 75.00] | 63.00 [51.00, 86.00] | 63.00 [54.75, 72.00] | 0.073 |
| High international normalised ratio | 1.30 [1.20, 1.70] | 1.80 [1.30, 2.50] | 1.20 [1.10, 1.30] | <0.001 |
| High d-dimer, (mg/L) | 1.85 [1.08, 8.93] | 9.65 [6.00, 10.00] | 1.20 [0.91, 1.91] | 0.022 |
| Low fibrinogen, (g/L) | 4.80 [3.10, 6.45] | 2.60 [1.70, 4.30] | 5.70 [4.30, 7.00] | <0.001 |
| High anti-Xa, (IU) | 0.25 [0.09, 0.38] | 0.10 [0.00, 0.28] | 0.26 [0.18, 0.43] | 0.024 |
| Mean anti-Xa, (IU) | 0.21 [0.05, 0.37] | 0.06 [0.00, 0.23] | 0.27 [0.11, 0.43] | 0.017 |
| Antithrombin, (%) | 68.00 [48.25, 85.25] | 42.00 [33.50, 55.00] | 78.50 [59.00, 90.25] | <0.001 |
| vWF antigen, (%) | 414.00 [344.00, 485.00] | 420.50 [336.00, 490.00] | 405.00 [353.00, 474.00] | 0.271 |
| vWF activity, (%) | 239.00 [188.00, 342.50] | 219.00 [195.50, 308.50] | 259.00 [187.50, 347.50] | 0.760 |
| vWF ratio | 0.62 [0.53, 0.73] | 0.60 [0.56, 0.70] | 0.64 [0.53, 0.76] | 0.972 |

**Table S3 Anti-coagulation Management**

|  | Overall (n = 221) | VA (n = 76) | VV (n = 145) | p |
| --- | --- | --- | --- | --- |
| **Daily Anticoagulation target, n (%)** |  |  |  |  |
| No infusion | 20 (9.4) | 15 (20.3) | 5 (3.6) | P <0.490 |
| Activated partial thromboplastin time < 45 seconds | 3 (1.4) | 1 (1.4) | 2 (1.4) |  |
| Activated partial thromboplastin time 50 - 70 seconds | 95 (44.8) | 27 (36.5) | 68 (49.3) |  |
| Activated partial thromboplastin time aPTT 60 - 80 seconds | 65 (30.7) | 14 (18.9) | 51 (37.0) |  |
| Other | 29 (13.7) | 17 (23.0) | 12 (8.7) |  |
| Very high activated partial thromboplastin time > 100 seconds | 76 (34.4) | 55 (30.9) | 18 (50.0) | 0.066 |
| **Heparin management and dosing, n (%)** |  |  |  |  |
| Heparin cessation for any reason | 84 (42.4) | 48 (68.6) | 36 (28.1) | <0.001 |
| Heparin ceased for bleeding or suspected bleeding | 28 (12.7) | 19 (25.0) | 9 (6.2) | 0.042 |
| Heparin ceased for “auto-anticoagulation” | 17 (7.7) | 14 (18.4) | 3 (2.1) | 0.032 |
| Heparin ceased for supra-therapeutic APTT* | 16 (7.2) | 6 (7.9) | 10 (6.9) | 0.970 |
| Heparin ceased for HITTS^@^ or HITT Suspicion | 1 (0.5) | 1 (1.3) | 0 (0.0) | 1.000 |
| Heparin ceased for disseminated intravascular coagulopathy | 4 (1.8) | 3 (3.9) | 1 (0.7) | 0.820 |
| Perceived bleeding risk e.g. thrombocytopenia | 3 (1.4) | 1 (1.3) | 2 (1.4) | 0.970 |
| Decannulated | 13 (5.9) | 9 (11.8) | 4 (2.8) | 0.012 |
| Not ceased just partial day, e.g. first day of support | 8 (3.6) | 7 (9.2) | 1 (0.7) | 0.013 |
| Daily heparin dose, IU (median [IQR]) | 24000 [9500, 36000] | 8500.00 [2500.00, 24000] | 28800 [17300, 40800] | 0.013 |
| Daily heparin dose, IU/kg/hr (median [IQR]) | 12.46 [6.82, 16.67] | 9.59 [2.05, 14.04] | 13.64 [8.74, 19.01] | 0.012 |
| Heparin infusion, hours per day (median [IQR]) | 24.00 [14.00, 24.00] | 14.00 [4.50, 24.00] | 24.00 [24.00, 24.00] | 0.005 |
| **Mean daily APTT, n (%)** |  |  |  |  |
| Within target range, n (%) | 90 (48.6) | 20 (35.1) | 70 (54.7) | 0.032 |
| Below target range, n (%) | 56 (30.3) | 18 (31.6) | 38 (29.7) | <0.001 |
| Above target range, n (%) | 44 (23.8) | 20 (35.1) | 24 (18.8) | 0.069 |

**APTT = activated partial thromboplastin time, ^@^HITTS = Heparin induced thrombotic thrombocytopenia, ^!^IQR = Interquartile range,*

**Supplementary Table 4 Calibrated Automated Thrombinogram (CAT) Results - Overall and by day of testing**

**Calibrated Automated Thrombinography – Overall Results**

| Calibrated Automated Thrombinography data, median [IQR]^*^ | VA ECMO | VV ECMO | P - value |
| --- | --- | --- | --- |
| Activated partial thromboplastin time, (s)^@^ | 52.80 [42.00, 73.10] | 56.50 [43.10, 66.10] | 0.015 |
| Unfractionated heparin level, (IU)^#^ | 0.07 [0.00, 0.27] | 0.21 [0.07, 0.38] | 0.450 |
| Lag time, (s) | 1.74 [0.00, 2.08] | 0.00 [0.00, 1.92] | 0.014 |
| Peak Height, (nmol/L) | 128.01 [0.00, 182.08] | 0.00 [0.00, 174.67] | 0.020 |
| Time to peak, (s) | 3.05 [0.00, 3.83] | 0.00 [0.00, 3.47] | 0.012 |
| Endogenous thrombin potential, (nmol/L min) | 570.03 [0.00, 1024.60] | 0.00 [0.00, 707.18] | 0.015 |
| Normalised lag time, (s) | 1.33 [0.00, 1.54] | 0.00 [0.00, 1.48] | 0.017 |
| Normalised peak height, (nmol/L) | 25.17 [0.00, 35.80] | 0.00 [0.00, 35.51] | 0.031 |
| Normalised time to peak, (s) | 1.27 [0.00, 1.55] | 0.00 [0.00, 1.41] | 0.015 |
| Normalised endogenous thrombin potential, (nmol/L min) | 28.45 [0.00, 50.68] | 0.00 [0.00, 37.32] | 0.022 |

**IQR = Interquartile Range, ^@^s = seconds, ^#^IU = international units*

**Day Zero**

| Calibrated Automated Thrombinography data, median [IQR]^*^ | Overall (n = 33) | VA (n = 12) | VV (n = 21) | P - value |
| --- | --- | --- | --- | --- |
| Activated partial thromboplastin time, (s)^@^ | 59.10 [43.70, 72.20] | 88.10 [53.55, 200.00] | 51.50 [40.10, 63.10] | 0.011 |
| Unfractionated heparin level, (IU)^#^ | 0.17 [0.02, 0.38] | 0.26 [0.00, 0.46] | 0.13 [0.03, 0.25] | 0.707 |
| Lag time, (s) | 1.23 [0.00, 1.90] | 0.61 [0.00, 1.91] | 1.74 [0.00, 1.90] | 0.906 |
| Peak Height, (nmol/L) | 37.20 [0.00, 204.31] | 18.60 [0.00, 138.62] | 67.40 [0.00, 257.20] | 0.238 |
| Time to peak, (s) | 2.38 [0.00, 3.29] | 1.19 [0.00, 3.37] | 2.72 [0.00, 3.29] | 0.969 |
| Endogenous thrombin potential, (nmol/L min) | 338.53 [0.00, 1006.90] | 169.26 [0.00, 647.12] | 393.48 [0.00, 1105.52] | 0.326 |
| Normalised lag time, (s) | 0.96 [0.00, 1.48] | 0.47 [0.00, 1.46] | 1.37 [0.00, 1.48] | 0.686 |
| Normalised peak height, (nmol/L) | 7.32 [0.00, 38.81] | 3.66 [0.00, 27.27] | 13.25 [0.00, 50.55] | 0.251 |
| Normalised time to peak, (s) | 0.98 [0.00, 1.41] | 0.49 [0.00, 1.39] | 1.14 [0.00, 1.41] | 0.865 |
| Normalised endogenous thrombin potential, (nmol/L min) | 16.72 [0.00, 49.33] | 8.36 [0.00, 31.73] | 19.82 [0.00, 54.34] | 0.349 |

**IQR = Interquartile Range, ^@^s = seconds, ^#^IU = international units*

**CAT Day 2**

| Calibrated Automated Thrombinography data, median [IQR]^*^ | Overall (n = 29) | VA (n = 10) | VV (n = 19) | P - value |
| --- | --- | --- | --- | --- |
| Activated partial thromboplastin time, (s)^@^ | 51.40 [41.90, 60.20] | 46.60 [40.32, 56.75] | 52.00 [43.55, 61.50] | 0.224 |
| Unfractionated heparin level, (IU)^#^ | 0.10 [0.03, 0.27] | 0.03 [0.00, 0.07] | 0.19 [0.08, 0.34] | 0.003 |
| Lag time, (s) | 1.77 [0.00, 2.12] | 1.82 [1.70, 2.03] | 0.00 [0.00, 2.12] | 0.325 |
| Peak Height, (nmol/L) | 50.92 [0.00, 180.65] | 173.88 [134.56, 213.35] | 0.00 [0.00, 54.59] | 0.006 |
| Time to peak, (s) | 3.06 [0.00, 4.34] | 3.23 [2.96, 4.18] | 0.00 [0.00, 4.22] | 0.242 |
| Endogenous thrombin potential, (nmol/L min) | 364.59 [0.00, 971.68] | 941.81 [595.75, 1178.67] | 0.00 [0.00, 403.70] | 0.004 |
| Normalised lag time, (s) | 1.33 [0.00, 1.58] | 1.40 [1.32, 1.51] | 0.00 [0.00, 1.62] | 0.164 |
| Normalised peak height, (nmol/L) | 10.09 [0.00, 37.42] | 33.84 [26.46, 41.26] | 0.00 [0.00, 11.21] | 0.010 |
| Normalised time to peak, (s) | 1.26 [0.00, 1.55] | 1.35 [1.24, 1.69] | 0.00 [0.00, 1.51] | 0.099 |
| Normalised endogenous thrombin potential, (nmol/L min) | 21.21 [0.00, 50.55] | 46.59 [29.52, 58.70] | 0.00 [0.00, 21.21] | 0.007 |

**IQR = Interquartile Range, ^@^s = seconds, ^#^IU = international units*

**CAT Day 4**

| Calibrated Automated Thrombinography data, median [IQR]^*^ | Overall (n = 22) | VA (n = 6) | VV (n = 16) | P value |
| --- | --- | --- | --- | --- |
| Activated partial thromboplastin time, (s)^@^ | 57.90 [41.03, 68.10] | 45.75 [41.03, 51.97] | 61.90 [47.95, 68.90] | 0.238 |
| Unfractionated heparin level, (IU)^#^ | 0.17 [0.07, 0.39] | 0.06 [0.01, 0.17] | 0.34 [0.12, 0.41] | 0.024 |
| Lag time, (s) | 0.00 [0.00, 1.80] | 1.80 [1.37, 2.41] | 0.00 [0.00, 0.35] | 0.038 |
| Peak Height, (nmol/L) | 0.00 [0.00, 134.23] | 134.15 [9.14, 181.28] | 0.00 [0.00, 33.56] | 0.131 |
| Time to peak, (s) | 0.00 [0.00, 3.17] | 3.17 [3.05, 4.20] | 0.00 [0.00, 0.67] | 0.038 |
| Endogenous thrombin potential, (nmol/L min) | 0.00 [0.00, 704.69] | 642.49 [176.07, 1045.05] | 0.00 [0.00, 176.17] | 0.047 |
| Normalised lag time, (s) | 0.00 [0.00, 1.46] | 1.39 [1.05, 1.79] | 0.00 [0.00, 0.55] | 0.049 |
| Normalised peak height, (nmol/L) | 0.00 [0.00, 28.70] | 25.81 [1.80, 35.65] | 0.00 [0.00, 13.19] | 0.168 |
| Normalised time to peak, (s) | 0.00 [0.00, 1.38] | 1.31 [1.26, 1.70] | 0.00 [0.00, 0.57] | 0.049 |
| Normalised endogenous thrombin potential, (nmol/L min) | 0.00 [0.00, 35.45] | 30.77 [8.70, 51.62] | 0.00 [0.00, 17.72] | 0.076 |

**IQR = Interquartile Range, ^@^s = seconds, ^#^IU = international units*

**CAT Day 6**

| Calibrated Automated Thrombinography data, median [IQR]^*^ | Overall (n = 14) | VA (n = 1) | VV (n = 13) | P - value |
| --- | --- | --- | --- | --- |
| Activated partial thromboplastin time, (s)^@^ | 58.85 [48.27, 70.62] | 83.10 [83.10, 83.10] | 56.50 [47.50, 66.80] | 0.172 |
| Unfractionated heparin level, (IU)^#^ | 0.32 [0.16, 0.41] | 0.32 [0.32, 0.32] | 0.31 [0.16, 0.43] | 0.901 |
| Lag time, (s) | 0.00 [0.00, 0.00] | 0.00 [0.00, 0.00] | 0.00 [0.00, 0.00] | 0.684 |
| Peak Height, (nmol/L) | 0.00 [0.00, 0.00] | 0.00 [0.00, 0.00] | 0.00 [0.00, 0.00] | 0.684 |
| Time to peak, (s) | 0.00 [0.00, 0.00] | 0.00 [0.00, 0.00] | 0.00 [0.00, 0.00] | 0.684 |
| Endogenous thrombin potential, (nmol/L min) | 0.00 [0.00, 0.00] | 0.00 [0.00, 0.00] | 0.00 [0.00, 0.00] | 0.684 |
| Normalised lag time, (s) | 0.00 [0.00, 0.00] | 0.00 [0.00, 0.00] | 0.00 [0.00, 0.00] | 0.671 |
| Normalised peak height, (nmol/L) | 0.00 [0.00, 0.00] | 0.00 [0.00, 0.00] | 0.00 [0.00, 0.00] | 0.671 |
| Normalised time to peak, (s) | 0.00 [0.00, 0.00] | 0.00 [0.00, 0.00] | 0.00 [0.00, 0.00] | 0.671 |
| Normalised endogenous thrombin potential, (nmol/L min) | 0.00 [0.00, 0.00] | 0.00 [0.00, 0.00] | 0.00 [0.00, 0.00] | 0.671 |

**IQR = Interquartile Range, ^@^s = seconds, ^#^IU = international units*

**CAT Decannulation**

| Calibrated Automated Thrombinography data, median [IQR]^*^ | Overall (n = 22) | VA (n = 9) | VV (n = 13) | P - value |
| --- | --- | --- | --- | --- |
| Activated partial thromboplastin time, (s)^@^ | 33.50 [29.70, 37.02] | 33.70 [30.60, 42.20] | 33.30 [29.70, 36.20] | 0.570 |
| Unfractionated heparin level, (IU)^#^ | 0.04 [0.02, 0.06] | 0.05 [0.01, 0.17] | 0.03 [0.02, 0.05] | 0.419 |
| Lag time, (s) | 2.12 [1.86, 2.44] | 2.03 [1.47, 2.24] | 2.25 [1.94, 2.49] | 0.151 |
| Peak Height, (nmol/L) | 305.38 [213.40, 352.16] | 157.57 [58.56, 316.90] | 322.22 [281.32, 363.76] | 0.049 |
| Time to peak, (s) | 3.69 [3.07, 4.07] | 3.71 [2.56, 4.09] | 3.67 [3.12, 4.01] | 0.713 |
| Endogenous thrombin potential, (nmol/L min) | 1057.21 [918.62, 1429.86] | 912.65 [352.02, 968.57] | 1279.93 [1015.61, 1561.73] | 0.010 |
| Normalised lag time, (s) | 1.60 [1.38, 1.84] | 1.54 [1.14, 1.73] | 1.71 [1.49, 1.94] | 0.239 |
| Normalised peak height, (nmol/L) | 60.57 [38.91, 66.97] | 30.98 [11.52, 62.32] | 64.99 [56.50, 70.94] | 0.053 |
| Normalised time to peak, (s) | 1.54 [1.25, 1.63] | 1.58 [1.06, 1.63] | 1.54 [1.30, 1.66] | 0.542 |
| Normalised endogenous thrombin potential, (nmol/L min) | 53.25 [44.41, 69.36] | 45.20 [17.44, 48.78] | 65.04 [56.95, 80.75] | 0.007 |

**IQR = Interquartile Range, ^@^s = seconds, ^#^IU = international units*

**Table S5 Thromboelastography Global Haemostasis** `

|  | Overall (n = 221) | VA (n = 76) | VV (n = 145) | p - value |
| --- | --- | --- | --- | --- |
| **Kaolin Assay (CK), median [IQR]** |  |  |  |  |
| R time (mins) | 20.20 [11.20, 40.75] | 15.40 [8.45, 30.90] | 26.20 [15.60, 42.00] | 0.002 |
| A10 ($^{\circ}$) | 39.85 [25.57, 64.32] | 0.00 [0.00, 0.00] | 42.30 [34.80, 66.10] | <0.001 |
| MA (mm) | 57.35 [41.50, 63.80] | 49.20 [24.52, 56.05] | 61.40 [55.58, 65.50] | 0.006 |
| G value | 6.72 [3.54, 8.81] | 4.85 [1.78, 6.38] | 7.96 [6.26, 9.50] | 0.009 |
| LY30 (%) | 0.00 [0.00, 0.00] | 0.00 [0.00, 0.00] | 0.00 [0.00, 0.00] | 0.454 |
| **Rapid TEG (CRT), median [IQR]** |  |  |  |  |
| Activated clotting time (s) | 116.00 [101.95, 134.70] | 125.30 [116.00, 200.20] | 116.00 [97.30, 120.50] | 0.006 |
| R time (mins) | 0.70 [0.55, 0.90] | 0.80 [0.70, 1.60] | 0.70 [0.50, 0.75] | 0.003 |
| A10 ($^{\circ}$) | 70.50 [66.10, 73.90] | 49.30 [47.60, 60.00] | 70.75 [67.75, 74.05] | 0.128 |
| MA (mm) | 68.30 [58.45, 72.15] | 56.55 [49.60, 67.03] | 70.40 [66.05, 73.85] | 0.009 |
| G value | 10.77 [7.04, 12.96] | 6.51 [4.92, 10.16] | 11.89 [9.72, 14.12] | 0.002 |
| LY30 (%) | 0.00 [0.00, 0.00] | 0.00 [0.00, 0.00] | 0.00 [0.00, 0.00] | 0.927 |
| **Kaolin with heparin (CKH), median [IQR]** |  |  |  |  |
| R time (mins) | 10.40 [8.25, 13.00] | 9.40 [8.35, 12.10] | 10.80 [8.25, 13.05] | 0.200 |
| A10 ($^{\circ}$) | 63.10 [54.60, 68.30] | 2.45 [1.62, 3.33] | 64.80 [58.70, 68.30] | 0.001 |
| MA (mm) | 64.70 [58.50, 68.95] | 55.55 [50.22, 63.70] | 67.20 [63.60, 69.35] | 0.023 |
| G value | 9.16 [7.05, 11.11] | 6.25 [5.04, 8.78] | 10.24 [8.74, 11.31] | 0.006 |
| **Functional fibrinogen (CFF), median [IQR]** |  |  |  |  |
| MA (mm) | 30.60 [18.50, 49.40] | 17.85 [12.25, 27.55] | 38.40 [28.75, 56.05] | 0.006 |
| FLEV (mg/ml) | 558.40 [337.55, 901.45] | 325.70 [223.55, 502.75] | 700.70 [524.25, 1022.80] | 0.014 |
| G value | 2.20 [1.14, 4.88] | 1.08 [0.69, 1.90] | 3.12 [2.01, 6.38] | 0.002 |

**Table S6 - Platelet Function tests VA ECMO versus VV ECMO Patients**

|  | Overall | VA | VV | p |
| --- | --- | --- | --- | --- |
| **TEG Heparinized kaolin with heparinise (HKH), median [IQR]** | |  |  |  |
| R time (mins) | 7.75 [6.50, 9.65] | 7.60 [6.53, 8.55] | 7.85 [6.50, 10.18] | 0.413 |
| A10 ($^{\circ}$) | 64.55 [53.99, 70.45] | 64.00 [58.55, 65.50] | 65.10 [52.02, 70.60] | 0.719 |
| MA (mm) | 66.75 [60.58, 69.00] | 60.15 [54.60, 66.17] | 67.55 [65.18, 70.00] | 0.028 |
| G value | 10.04 [7.69, 11.13] | 7.55 [6.01, 9.80] | 10.41 [9.36, 11.67] | 0.012 |
| LY30% | 0.00 [0.00, 0.00] | 0.00 [0.00, 0.20] | 0.00 [0.00, 0.00] | 0.378 |
| **TEG Activator F, median [IQR]** |  |  |  |  |
| ACT F MA (mm) | 22.70 [16.22, 34.40] | 14.55 [6.72, 22.68] | 27.70 [19.68, 38.03] | <0.001 |
| ACT F G value | 1.47 [0.97, 2.63] | 0.85 [0.36, 1.47] | 1.92 [1.22, 3.07] | <0.001 |
| **TEG ADP (adenosine-5'-diphosphate), median [IQR]** | |  |  |  |
| ADP MA (mm) | 49.15 [22.22, 64.18] | 29.05 [17.42, 47.62] | 56.85 [32.58, 65.15] | 0.007 |
| ADP G value | 4.84 [1.43, 8.96] | 2.04 [1.06, 4.55] | 6.58 [2.42, 9.35] | 0.009 |
| **Multiplate Aggregometry, median [IQR]** |  |  |  |  |
| ADP Aggregation units | 48.00 [18.25, 86.75] | 20.00 [12.00, 47.00] | 64.00 [24.50, 98.75] | 0.011 |
| TRAP Aggregation units | 114.50 [61.50, 143.50] | 66.00 [52.00, 97.25] | 125.00 [106.25, 157.00] | 0.063 |

**Table S7 Bleeding Event Summary**

|  | Overall (n = 25) | VA ECMO (n = 9) | VV ECMO (n = 16) | P - value |
| --- | --- | --- | --- | --- |
| Day of bleeding, median, [IQR] | 1.20 [0.30, 6.50] | 0.30 [0.00, 0.50] | 4.30 [1.10, 7.15] | 0.001 |
| **Bleeding Site, n (%)** |  |  |  |  |
| Cannula | 8 (33.3) | 5 (55.6) | 3 (20.0) | 0.421 |
| Surgical site e.g. on open cut-down | 7 (29.2) | 2 (22.2) | 5 (33.3) |  |
| Central nervous system bleeding | 2 (8.3) | 0 (0.0) | 2 (13.3) |  |
| Ear nose and throat e.g. nasal bleeding | 2 (8.3) | 0 (0.0) | 2 (13.3) |  |
| Haematuria | 3 (12.5) | 1 (11.1) | 2 (13.3) |  |
| Chest drain | 1 (4.2) | 0 (0.0) | 1 (6.7) |  |
| Liver bleeding confirmed on imaging | 1 (4.2) | 1 (11.1) | 0 (0.0) |  |
| Cannula bleeding requiring transfusion or surgical intervention, n (%) | 7 (87.5) | 5 (100.0) | 2 (66.7) | 0.375 |
| **GI Bleeding Type, n (%)** |  |  |  |  |
| Coffee Ground vomitus | 2 (28.6) | 2 (100.0) | 0 (0.0) | 0.048 |
| Malaena | 1 (14.3) | 0 (0.0) | 1 (20.0) |  |
| Upper GI frank blood | 4 (57.1) | 0 (0.0) | 4 (80.0) |  |
| **Bleeding by BARC Bleeding Criteria, n (%)** |  |  |  |  |
| Type 1 - No attention needed | 7 (32.0) | 3 (33.3) | 4 (25.0) | 0.548 |
| Type 2 – Overt actionable bleeding but Hb <30 drop | 11 (44.0) | 3 (33.3) | 8 (50.0) |  |
| Type 3a - Overt bleeding with Hb >30 or 3 units PRBC – 5PRBC | 4 (16.0) | 2 (22.2) | 2 (12.5) |  |
| Type 3b - Overt bleeding with Hb >50 or escalation of vasopressors or surgical intervention or tamponade | 1 (4.0) | 1 (11.1) | 0 (0.0) |  |
| Type 5 Fatal Bleeding | 2 (8.0) | 0 (0.0) | 2 (12.5) |  |

**Supplementary Table S8 – Circuit Variables**

| Daily values | Overall (n = 221) | VA (n = 76) | VV (n = 145) | P value |
| --- | --- | --- | --- | --- |
| Temperature (median [IQR]) | 36.50 [36.00, 36.80] | 36.50 [36.00, 36.80] | 36.50 [36.10, 36.80] | 0.154 |
| Lowest daily pump flow (median [IQR]) | 2.97 [2.42, 3.57] | 2.70 [2.20, 3.05] | 3.04 [2.51, 3.88] | 0.004 |
| Highest daily pump flow (median [IQR]) | 3.29 [2.93, 4.13] | 3.24 [2.89, 3.79] | 3.44 [2.95, 4.51] | 0.088 |
| Mean daily pump flow (median [IQR]) | 3.10 [2.67, 3.77] | 2.96 [2.57, 3.33] | 3.15 [2.80, 4.18] | 0.019 |
| Lowest daily pump speed (median [IQR]) | 2705.00 [2434.00, 3150.00] | 2760.00 [2440.00, 2970.00] | 2697.50 [2377.50, 3180.00] | 0.695 |
| Highest daily pump speed (median [IQR]) | 2900.00 [2547.00, 3270.00] | 2895.00 [2625.00, 3259.00] | 2900.00 [2506.00, 3310.00] | 0.773 |
| Mean daily pump speed (median [IQR]) | 2832.00 [2510.25, 3180.00] | 2845.00 [2567.50, 3092.50] | 2803.50 [2477.50, 3200.62] | 0.710 |
| Lowest daily pre-membrane pressure (median [IQR]) | 152.00 [121.00, 201.00] | 165.00 [141.00, 186.00] | 143.00 [112.25, 204.00] | 0.681 |
| Highest daily pre-membrane pressure (median [IQR]) | 181.00 [144.50, 228.00] | 192.00 [170.00, 232.00] | 165.00 [135.25, 226.00] | 0.519 |
| Mean daily pre-membrane pressure (median [IQR]) | 168.00 [134.50, 209.25] | 176.00 [157.00, 206.50] | 155.75 [125.12, 210.88] | 0.403 |
| Lowest post-membrane pressure (median [IQR]) | 136.00 [110.00, 188.00] | 151.00 [130.00, 173.00] | 125.00 [94.25, 189.00] | 0.438 |
| Highest post-membrane pressure (median [IQR]) | 165.00 [130.50, 215.00] | 180.00 [154.00, 220.00] | 150.00 [119.00, 210.50] | 0.364 |
| Mean post-membrane pressure (median [IQR]) | 151.00 [119.75, 193.75] | 163.50 [145.50, 193.00] | 137.50 [108.62, 197.75] | 0.403 |
| Transmembrane pressure (median [IQR]) | -16.00 [-21.50, -12.00] | -14.50 [-17.50, -9.50] | -17.50 [-23.88, -12.12] | 0.848 |
| Clots on circuit noted, n (%) | 89 (41.4) | 30 (41.1) | 59 (41.5) | 0.980 |

**Table S9 – Cannulation details**

|  | VA (n = 17) | VV (n = 22) |
| --- | --- | --- |
| **VA Access** |  |  |
| Central VA access n, (%) | 1 (5.9) |  |
| 21Fr Access Cannula | 1 (5.9) |  |
| 23Fr Access Cannula | 5 (29.4) |  |
| 25Fr Access Cannula | 10 (58.8) |  |
| 29Fr Access Cannula | 1 (5.9) |  |
| **VA Access cannulae technique** |  |  |
| Landmark | 3 (18.8) |  |
| Ultrasound guides | 11 (68.8) |  |
| Cut down | 2 (12.5) |  |
| **VA Return cannulae** |  |  |
| Left femoral artery return | 7 (41.2) |  |
| Return cannulae size |  |  |
| 15Fr | 1 (6.2) |  |
| 17Fr | 9 (56.2) |  |
| 19Fr | 5 (31.2) |  |
| 21Fr | 1 (6.2) |  |
| **VA return cannulae technique** |  |  |
| Landmark | 1 (5.9) |  |
| Ultrasound | 13 (76.5) |  |
| Cutdown | 2 (11.8) |  |
| Open surgical | 1 (5.9) |  |
| Backflow cannulae implemented | 16 (94.1) |  |
| **VV Configuration** |  |  |
| Bicaval Dual-Lumen (Avalon) |  | 10 (45.5) |
| Femoral vein – femoral vein |  | 5 (22.7) |
| Femoral vein – jugular vein |  | 7 (31.8) |

**Table S10 – Subgroup Analysis of daily standard lab tests of VA ECMO patients ECPR status**

|  | Overall | VA non-ECPR | VA ECPR | P value |
| --- | --- | --- | --- | --- |
| n | 76 | 37 | 39 |  |
| Low Ionised calcium (median [IQR]) | 1.07 [1.02, 1.13] | 1.08 [1.05, 1.12] | 1.07 [1.00, 1.13] | 0.655 |
| High lactate (median [IQR]) | 2.60 [1.40, 6.50] | 1.60 [1.20, 2.92] | 6.20 [2.50, 8.90] | 0.028 |
| High urea (median [IQR]) | 8.00 [5.20, 10.30] | 7.95 [5.02, 11.60] | 8.10 [5.70, 9.40] | 0.758 |
| Creatinine high (median [IQR]) | 104.00 [84.00, 172.00] | 104.00 [86.75, 219.50] | 101.00 [82.00, 172.00] | 0.747 |
| Bilirubin high (median [IQR]) | 26.00 [16.00, 58.00] | 41.00 [13.00, 69.75] | 23.00 [16.00, 35.00] | 0.938 |
| Lactate dehydrogenase high (median [IQR]) | 896.50 [482.75, 2321.25] | 873.00 [479.00, 1836.00] | 1408.00 [494.00, 2588.00] | 0.510 |
| Haemoglobin low (median [IQR]) | 84.00 [76.00, 100.00] | 89.50 [78.75, 104.25] | 80.00 [76.00, 86.00] | 0.424 |
| Platelet low (median [IQR]) | 89.00 [59.00, 130.00] | 113.00 [81.50, 160.00] | 69.00 [50.00, 102.00] | 0.242 |
| Plasma free hemoglobin (median [IQR]) | 4.00 [2.50, 5.50] | 4.50 [2.50, 8.62] | 3.00 [2.25, 5.00] | 0.609 |
| aPTT high (median [IQR]) | 63.00 [51.00, 86.00] | 62.50 [52.00, 77.00] | 64.00 [50.00, 200.00] | 0.155 |
| International normalised ratio high (median [IQR]) | 1.80 [1.30, 2.50] | 1.75 [1.30, 2.52] | 1.80 [1.30, 2.50] | 0.937 |
| Fibrinogen high (median [IQR]) | 2.75 [21.8, 4.53] | 4.15 [2.62 – 5.88] | 2.05 [1.80 – 2.60] | 0.014 |
| Anti-FXa high (median [IQR]) | 0.10 [0.00, 0.28] | 0.08 [0.02, 0.26] | 0.10 [0.00, 0.28] | 0.418 |
| Antithrombin (median [IQR]) | 42.00 [33.50, 55.00] | 43.00 [34.00, 54.50] | 41.50 [29.75, 55.00] | 0.524 |
| vWF antigen (median [IQR]) | 420.50 [336.00, 490.00] | 469.50 [437.25, 644.00] | 336.00 [302.00, 352.75] | 0.599 |
| vWF activity (median [IQR]) | 219.00 [195.50, 308.50] | 341.00 [219.00, 375.00] | 203.50 [188.00, 226.25] | 0.017 |
| vWF ratio (median [IQR]) | 0.60 [0.56, 0.70] | 0.58 [0.48, 0.65] | 0.66 [0.60, 0.71] | 0.085 |

vWF – von Willebrand’s Factor

**Table S11 – Subgroup Analysis of daily standard lab tests non-ECPR VA patients compared with VV patients**

|  | Overall | VA (non ECPR) | VV | p |
| --- | --- | --- | --- | --- |
|  | 182 | 37 | 145 |  |
| Ionised calcium low (median [IQR]) | 1.11 [1.05, 1.18] | 1.08 [1.05, 1.12] | 1.12 [1.05, 1.20] | 0.181 |
| Lactate high (median [IQR]) | 1.50 [1.20, 1.90] | 1.60 [1.20, 2.92] | 1.50 [1.20, 1.80] | 0.028 |
| Urea high (median [IQR]) | 12.00 [8.20, 17.85] | 7.95 [5.02, 11.60] | 12.65 [9.28, 19.52] | 0.039 |
| Creatine high (median [IQR]) | 104.00 [78.00, 164.00] | 104.00 [86.75, 219.50] | 103.00 [77.00, 160.00] | 0.417 |
| Bilirubin high (median [IQR]) | 11.00 [7.00, 27.00] | 41.00 [13.00, 69.75] | 10.00 [7.00, 18.25] | 0.017 |
| Lactate dehydrogenase high (median [IQR]) | 469.00 [293.00, 758.00] | 873.00 [479.00, 1836.00] | 432.00 [292.50, 627.50] | 0.004 |
| Haemoglobin low (median [IQR]) | 85.00 [77.00, 97.00] | 89.50 [78.75, 104.25] | 83.00 [77.00, 95.00] | 0.482 |
| Platelet low (median [IQR]) | 176.00 [103.00, 216.00] | 113.00 [81.50, 160.00] | 186.00 [129.00, 225.00] | 0.353 |
| Plasma hemoglobin (median [IQR]) | 3.00 [2.50, 5.50] | 4.50 [2.50, 8.62] | 3.00 [2.50, 5.50] | 0.397 |
| aPTT high (median [IQR]) | 63.00 [53.00, 73.00] | 62.50 [52.00, 77.00] | 63.00 [54.00, 72.00] | 0.390 |
| International normalised ration high (median [IQR]) | 1.20 [1.10, 1.40] | 1.75 [1.30, 2.52] | 1.20 [1.10, 1.30] | 0.003 |
| Anti-FXa high (median [IQR]) | 0.26 [0.08, 0.42] | 0.08 [0.02, 0.26] | 0.27 [0.12, 0.45] | 0.082 |
| antithrombin (median [IQR]) | 72.50 [53.75, 87.00] | 43.00 [34.00, 54.50] | 78.50 [59.00, 90.25] | 0.003 |
| vWF antigen (median [IQR]) | 438.00 [378.00, 520.00] | 469.50 [437.25, 644.00] | 405.00 [353.00, 474.00] | 0.267 |
| vWF activity (median [IQR]) | 263.50 [202.25, 353.75] | 341.00 [219.00, 375.00] | 259.00 [187.50, 347.50] | 0.268 |
| vWF ratio (median [IQR]) | 0.60 [0.50, 0.74] | 0.58 [0.48, 0.65] | 0.64 [0.53, 0.76] | 0.490 |

vWF – von Willebrand’s Factor


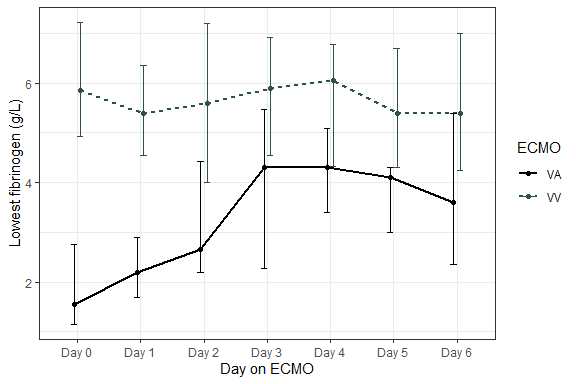


**Figure S1:** Daily Fibrinogen levels (lowest recorded on that day) during first week of ECMO support


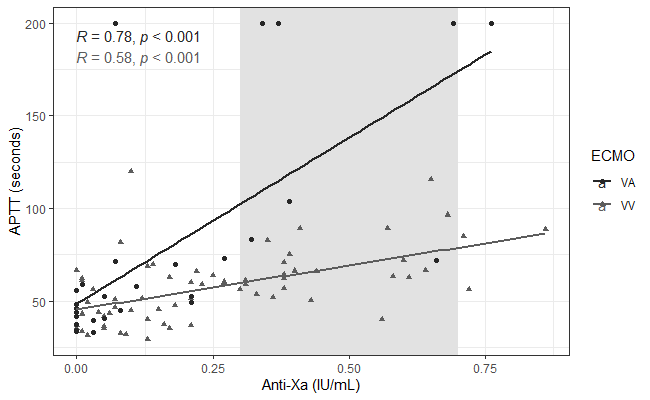


**Figure S2:** On matched sampling of APTT and Anti-Xa levels taken at time of calibrated automated thrombinography, the APTT level with the corresponding Anti-Xa level is presented by VV and VA patients. In VV patients a closer relationship between Anti-Xa levels and APTT levels is depicted. In VA patients a more disparate non-significant relationship is present. In particular note five separate recordings of APTT levels above 200 seconds corresponding with a wide range of Anti-Xa levels.


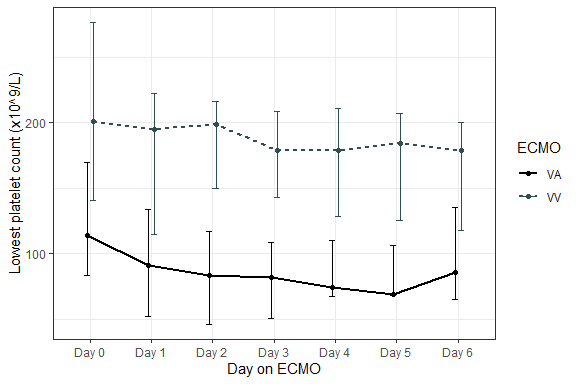


**Figure S3:** Daily Fibrinogen levels (lowest recorded on that day) during first week of ECMO support


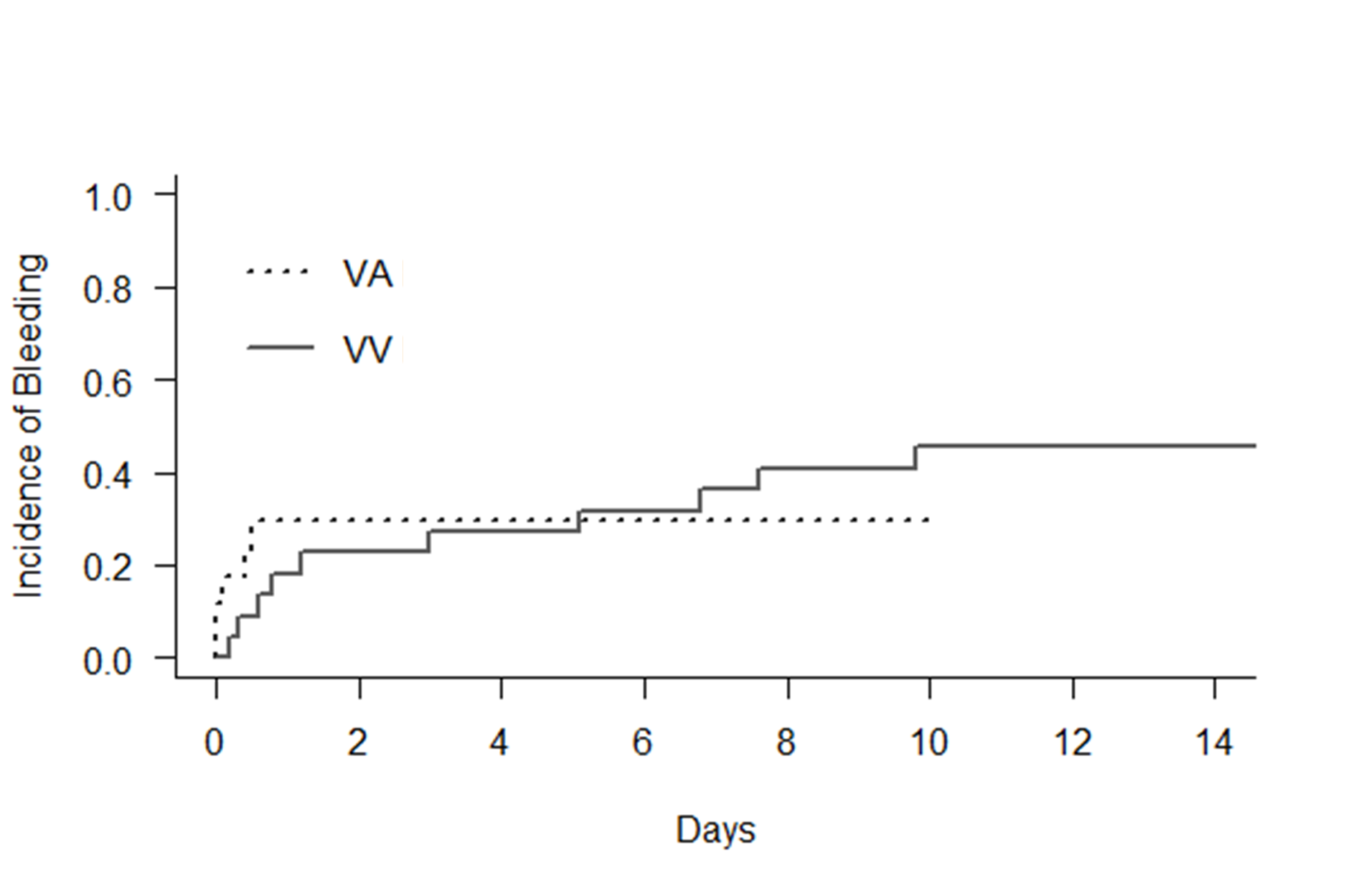


**Figure S3:** Cumulative incidence of bleeding events during ECMO run


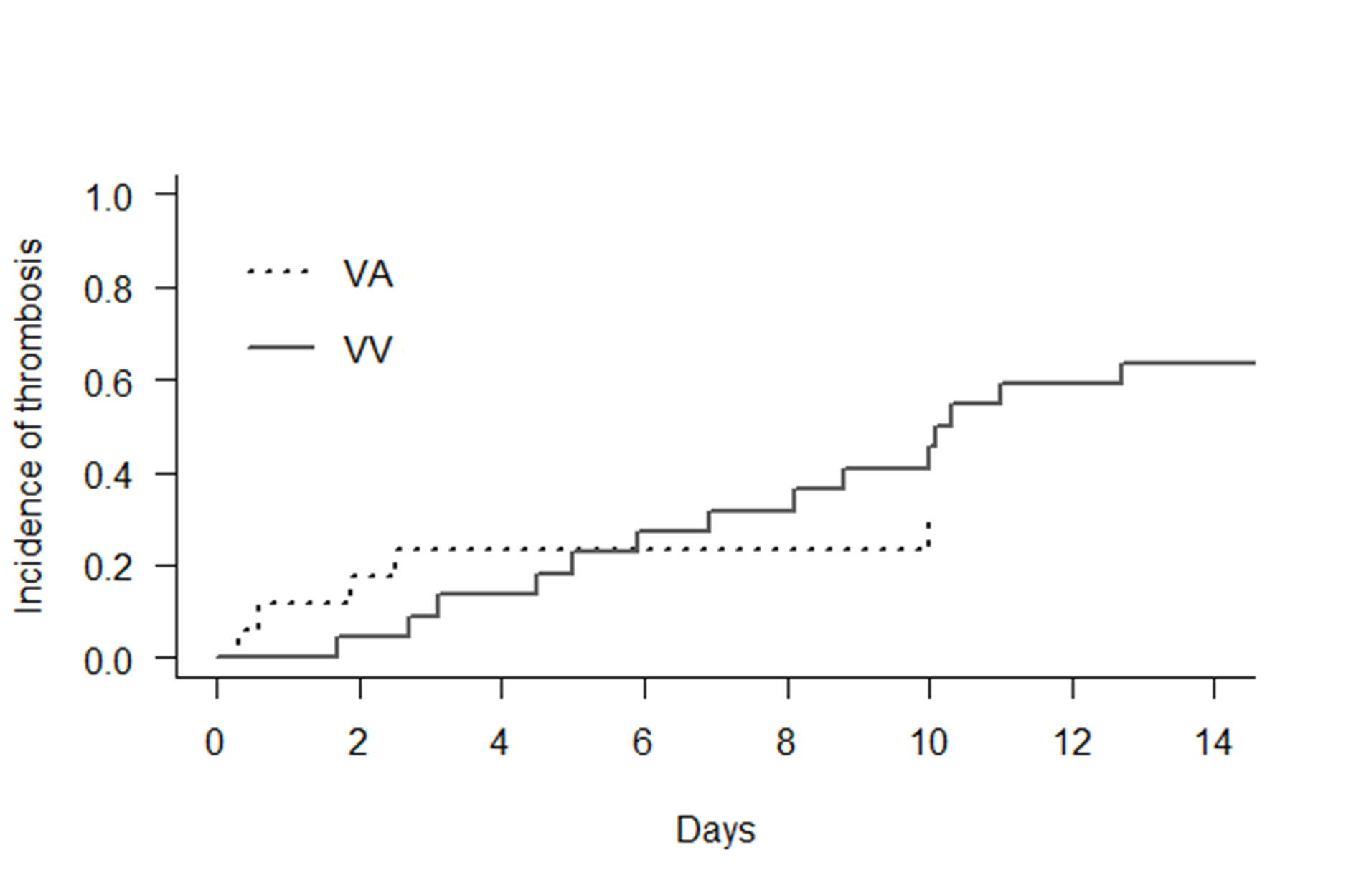


**Figure S4:** Cumulative incidence of thrombotic events during ECMO run
